# Rapid Analysis of the Chemical Composition of Xiaoban Kangfu Capsules Based on UHPLC-Q-Exactive Orbitrap MS/MS Combined with Molecular Networks

**DOI:** 10.3390/ph19030459

**Published:** 2026-03-11

**Authors:** Xia Luo, Yuehan Liao, Ting Qing, Jihui Zhao, Wei Cai

**Affiliations:** 1 School of Pharmacy, Ningxia Medical University, Yinchuan 750004, China; 241370410442@nxmu.edu.cn; 2 School of Pharmaceutical Sciences, Hunan University of Medicine, Huaihua 418000, China; houyuezuigege@gmail.com (Y.L.); linqing@csu.edu.cn (T.Q.)

**Keywords:** characterization, molecular networking, XBKF capsules, UHPLC-Q-Exactive Orbitrap mass spectrometry

## Abstract

**Background/Objectives:** Natural medicine analysis remains challenging due to chemical diversity. To the best of our knowledge, the comprehensive identification of multiple chemical constituents in Xiaoban Kangfu (XBKF) capsules has not been reported. Therefore, a combined approach utilizing ultra-high-performance liquid chromatography quadrupole-Exactive Orbitrap mass spectrometry (UHPLC-Q-Exactive Orbitrap MS) and molecular network analysis needs to be developed to comprehensively characterize the chemical constituents of XBK capsules in heat-clearing and toxin-eliminating granules, thereby enhancing annotation accuracy and enabling visualization. **Methods:** Firstly, chromatographic and mass spectrometry conditions were optimized to achieve good separation and a rich signal response. Subsequently, the literature searches, database consultations, and reference standards were employed to enhance annotation reliability. Finally, the raw data acquired under optimized conditions were uploaded to Global Natural Products Social (GNPSs), enabling component visualization by linking precursor ions of similar structural features with identical colors. **Results:** A total of 170 compounds were identified from this medicinal resource for the first time, including 50 flavonoids, 34 phenolic acids, 16 terpenoids, 14 quinones, 14 organic acids, eight coumarins, ive carbohydrates, and 29 other compounds. **Conclusions:** This study establishes a robust UHPLC-Q-Exactive Orbitrap MS/MS strategy for the comprehensive chemical profiling of XBKF capsules. The use of the presented validated analytical method for the comprehensive quality control of XBKF capsules is highly promising, offering fast, highly sensitive, and reliable analysis.

## 1. Introduction

Traditional Chinese medicine (TCM) formulas, composed of one or more drugs of plant origin, have been used as major remedies in China and other Asian countries for thousands of years with reliable efficacy [1]. XBKF capsules, composed of seven herbs including *Fructus mori*, *Salvia miltiorrhiza Bunge*, *Tribulus terrestris* L., *Typhonium giganteum Engl.*, *Eclipta prostrata*, *Glycyrrhiza uralensis*, and *Fallopia multiflora,* have nourishing yin [2,3] and blood circulation promotion effects, benefiting the liver and enhancing complexion, removing blood stasis and freckles [4], and improving microcirculation [5]. Clinically, they are widely used to treat vitiligo, premature graying of hair, facial freckles, and melasma, making them an effective compound for treating related skin conditions [6,7]. Although XBKF capsules are widely used in clinical practice, due to their complex composition, their comprehensive chemical information about them remains unclear. Therefore, the development of a systematic strategy for the rapid detection and identification of constituents in XBKF capsules is necessary.

Multiple approaches have been used in an attempt to explore the chemical components of TCM prescriptions. Large-scale TCM analyses can be performed thanks to ultra-high-performance liquid chromatography (UHPLC) technology advancements that have emerged in parallel with the development of mass spectrometry (MS). Ultra-high-performance liquid chromatography–mass spectrometry (UHPLC-MS/MS) has emerged as a highly sensitive and powerful instrument in recent years, as it provides precise information on ion precursors and fragment ions from MS/MS. This is beneficial for improving the authenticity of the characterization of small- and medium-sized molecules in mixtures [8,9,10,11]. However, differentiating structural isomers remains a challenge due to their minor spectral and structural differences [12]. Recently, UHPLC-Q-Exactive Orbitrap MS has emerged as a prominent tool for the rapid screening and characterization of chemical components [13,14]. This technology can realize the simultaneous and rapid identification of multiple components, even in the absence of reference standards, rendering it an exceptionally effective tool for detecting and identifying chemical components in traditional Chinese medicine [15].

Recently, GNPS (https://gnps.ucsd.edu) molecular networking (MN) was proven to be a powerful tool for MS data processing, based on MS/MS spectral similarity, which was introduced for the rapid recognition of known compounds and efficient elucidation of unreported ones [16]. Researchers use this technique extensively to investigate natural products, metabolomics, foodborne hazards, and clinical medicine [17]. This outcome highlights the power of GNPS as an invaluable tool for discovering compounds from natural sources.

To the best of our knowledge, the comprehensive identification of multiple chemical constituents in the XBKF capsule has not been reported in the literature. In this study, we present a comprehensive analysis of the major chemical components in XBKF capsules using UHPLC-Q Exactive-Orbitrap-MS, combined with the molecular network method, to rapidly analyze complex components in XBKF. This study facilitated the first comprehensive identification of 170 chemical constituents, comprising 50 flavonoids, 31 phenolic acids, 16 terpenoids, 14 quinones, 14 organic acids, eight coumarins, five carbohydrates, three phenylpropanoids, and 29 other compounds, providing in-depth knowledge and offering valuable information regarding quality control and future pharmacological studies.

## 2. Results

### 2.1. Characterization of the Chemical Components in XBKF Capsules Using UHPLC-Q-Exactive Orbitrap Based on GNPS

In this study, we identified a total of 170 compounds, which included 50 flavonoids, 34 phenolic acids, 16 terpenoids, 14 quinones, 14 organic acids, eight coumarins, five sugars, and 29 additional compounds. Detailed information regarding these components, such as peak number, retention time (Rt), precise molecular ions, chemical formula, mass error (±5 ppm), fragment ions, and compound names, is presented in Table 1 and Supplementary Table S1. The high-resolution extraction ion chromatograms of XBKF capsules, captured in both positive- and negative-ion modes, are illustrated in Figure S1. Furthermore, the original data from both ionic modes were processed using GNPS to differentiate isomers with similar MS^2^ spectra, facilitating the clustering and annotation of the same types of compounds. Consequently, the molecular network diagram of XBKF capsules was generated based on the similarity of the MS/MS spectra.

#### 2.1.1. Identification of Flavonoids

**Figure 1:**
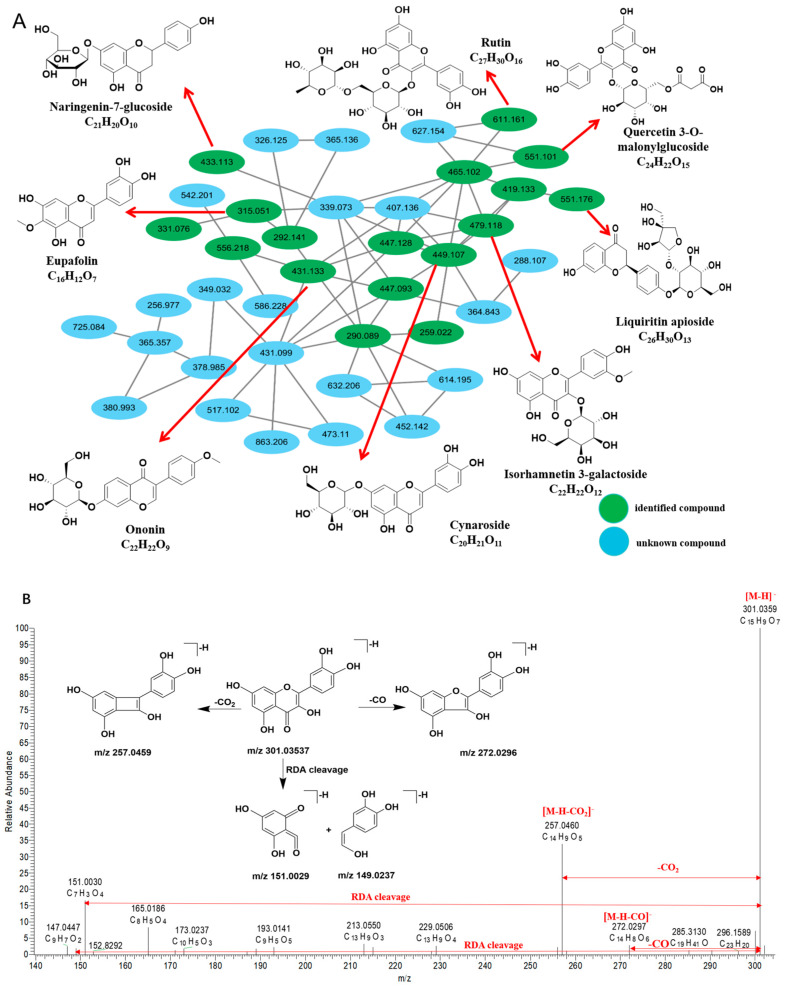
Flavonoid structure based on MS^2^ spectral analysis. (**A**) MN of flavonoid compounds based on their characteristics. (**B**) The MS/MS fragmentation pathways of quercetin in the extract of XBKF capsules.

This study identified 50 flavonoid compounds in XBKF capsules, comprising 15 flavonoids, 10 isoflavones, 11 flavonols, nine dihydroflavonoids, and five chalcones. MN was constructed based on UHPLC-Q-Exactive Orbitrap data (Figure 1).

Peak 45, the fragment ion peak at *m*/*z* 289.07176, is observed in the secondary mass spectrometry analysis. Based on the OTCML database and data reported by Chen et al. (2024), Yang et al. (2025) and Gevrenova et al. (2025) [18,19,20], the MS/MS fragmentation of catechin exhibited fragment ions at *m*/*z* 245.0822 [M-H-CO_2_]^−^, *m*/*z* 203.0711 [M-H-CO_2_-C_2_H_2_O]^−^, *m*/*z* 179.0345 [M-H-C_6_H_6_O_2_]^−^, and *m*/*z* 125.0235 [M-H-C_9_H_8_O_3_]^−^. In the case of peak 50, the excimer ion peak at *m*/*z* 417.1185 [M + H]^+^ is identified in positive-ion mode. This ion loses a neutral fragment, C_4_H_8_O_4_, resulting in the fragment ion at *m*/*z* 297.0744 [M + H-C_17_H_13_O_5_]^+^. After comparison with the OTCML and data reported by Grayer et al. (2000) [21], it was concluded that peak 50 corresponds to Puerarin, with the molecular formula C_21_H_20_O_9_.

Peak 59, observed at a retention time of 7.46 in negative-ion mode, exhibits an excimer ion peak corresponding to (C_15_H_9_O_7_^−^, *m*/*z* 301.03537) [M-H]^−^. The progressive loss of small molecules, specifically CO_2_ and CO, from the excimer ions yields fragment ions at *m*/*z* 257.0459 (C_14_H_9_O_5_^−^) and *m*/*z* 273.0407 (C_14_H_9_O_6_^−^). The B ring of the catechol structure experiences specific fragmentation, producing characteristic ions at *m*/*z* 151.0029 (C_7_H_3_O_4_^−^) and *m*/*z* 149.0237 (C_8_H_5_O_3_^−^). By comparing the data with the standards, OTCML database, and data reported by Chen et al. (2024) [18], peak 59 was identified as quercetin. The MS/MS fragmentation pathways are shown in Figure 1B.

The excimer ion peak for peak 71 is *m*/*z* 419.13365 [M + H]^+^. A notable feature of secondary mass spectrometry is the loss of one molecule of Glu, resulting in 257.0797 [M + H-Glu]^−^, *m*/*z* (C_15_H_13_O_4_^+^). Further loss of H_2_O (18.0101 Da) produces *m*/*z* 239.0694 (C_15_H_11_O_3_^+^). Through RDA cleavage, a pair of complementary characteristic ions is generated: ring A at *m*/*z* 147.0434 (C_9_H_7_O_2_^+^) and ring B at *m*/*z* 137.0228 (C_7_H_5_O_3_^+^). Comparison with GNPS and data reported by Pan et al. (2025) [22] allows for the compound at peak 71 to be identified as liquiritin.

In positive-ion mode, peak 75 exhibits a quimer ion peak corresponding to the precursor ion [M + H]^+^. At *m*/*z* 611.16066 (C_27_H_31_O_16_^+^), the glycosidic bond cleaves first, resulting in the loss of the entire rutin glycogroup (Rha + Glu). This process generates fragment ions at *m*/*z* 303.0485 (C_15_H_11_O_7_^+^). Additionally, oxygen-containing fragments at *m*/*z* 85.0286 (C_4_H_5_O_2_^+^) are formed from Rha following multiple dehydrations and carbon–carbon bond cleavages, along with ions at *m*/*z* 71.0495 (C_4_H_7_O^+^) resulting from the loss of small molecules. These fragments were compared with data from OTCML, GNPS, and the data reported by Wang et al. (2015) [23] and controlled substances. Consequently, it was determined that peak 75 corresponds to rutin, with the molecular formula C_27_H_30_O_16_.

Peak 77 displays an excimer ion peak identified as the precursor ion [M + H]^+^ at *m*/*z* 317.06557 (C_16_H_13_O_7_^+^). This ion undergoes cleavage through the RDA reaction, yielding a fragment ion at *m*/*z* 153.0162 (C_7_H_5_O_4_^+^). The carbon–oxygen bond in the methoxy group experiences homolytic cleavage, resulting in the loss of a neutral CH_3_ group and generating fragment ions at *m*/*z* 317.06557 (C_16_H_13_O_7_^+^), which possess an ortho-substituted structure of 3′-methoxy-4′-hydroxyl on its B ring. The loss of one methanol (CH_3_OH) produces a fragment ion at *m*/*z* 285.0382 (C_15_H_9_O_6_^+^). Therefore, by comparing its retention time and fragment ions with the data reported by Liu et al. (2024) [24], standard, and OTCML, it can be conclusively characterized that peak 77 corresponds to isorhamnetin, with the molecular formula C_16_H_12_O_7._

The excimer ion peak of peak 99 in the positive-ion mode is detected at *m*/*z* 551.17591 (C_26_H_31_O_13_^+^). The loss of one molecule each of Rha and Glu results in the formation of *m*/*z* 257.0800 (C_15_H_13_O_4_^+^). The cleavage patterns and molecular characteristics of this compound closely resemble those of glycyrrhizoside. By the data from OTCML, PubChem, and GNPS, peak 99 is identified as liquiritin apioside.

Peak 102, which has a retention time of 12.10 min, is also observable in the positive-ion mode. The excimer ion peak for this compound has a mass-to-charge ratio of *m*/*z* 419.13365, with secondary fragment ions at *m*/*z* 257, 137, and 147. The cleavage patterns and molecular characteristics of peak 102 align with those of isoliquiritin. Combined with analysis from GNPS and data reported by Pan et al. (2025) [22], this compound was identified as isoliquiritin.

Peak 122, with a retention time of 14.93 in negative-ion mode, exhibits an excimer ion peak corresponding to the precursor ion [M-H]^−^ at *m*/*z* 271.06119 (C_15_H_11_O_5_^−^). Fragment ions *m*/*z* 151.0030 (C_7_H_3_O_4_^−^) and *m*/*z* 119.0493 (C_8_H_7_O^−^) are generated through the retro-Diels–Alder (RDA) pyrolysis reaction. The *m*/*z* 151.0030 (C_7_H_3_O_4_^−^) ions can undergo rearrangement and subsequent cleavage to yield *m*/*z* 107.0128 (C_6_H_3_O_2_^−^). The *m*/*z* 119.0493 (C_8_H_7_O^−^) ion, which loses C_2_H_2_, serves as a characteristic ion of the B ring and further cleaves to produce *m*/*z* 93.0335 (C_6_H_5_O^−^). Based on comparisons with database entries and data reported by Zhu et al. (2022) [25], along with established mass spectrometry cleavage rules, it is concluded that peak 122 corresponds to naringenin, with the molecular formula C_15_H_12_O_5_.

Peak 157, also in negative-ion mode, shows an excimer ion peak for the precursor ion [M-H]^−^ at *m*/*z* 269.04554 (C_15_H_9_O_5_^−^). This quimer ion undergoes a series of neutral losses of small molecules. The loss of CO results in the fragment ion *m*/*z* 241.0509 (C_14_H_9_O_4_^−^), while the loss of CO_2_ generates the fragment ion *m*/*z* 225.0558 (C_14_H_9_O_3_^−^). It is determined that peak 157 corresponds to baicalein, with the molecular formula C_15_H_10_O_5._

#### 2.1.2. Identification of Phenolic Acid Compounds

**Figure 2:**
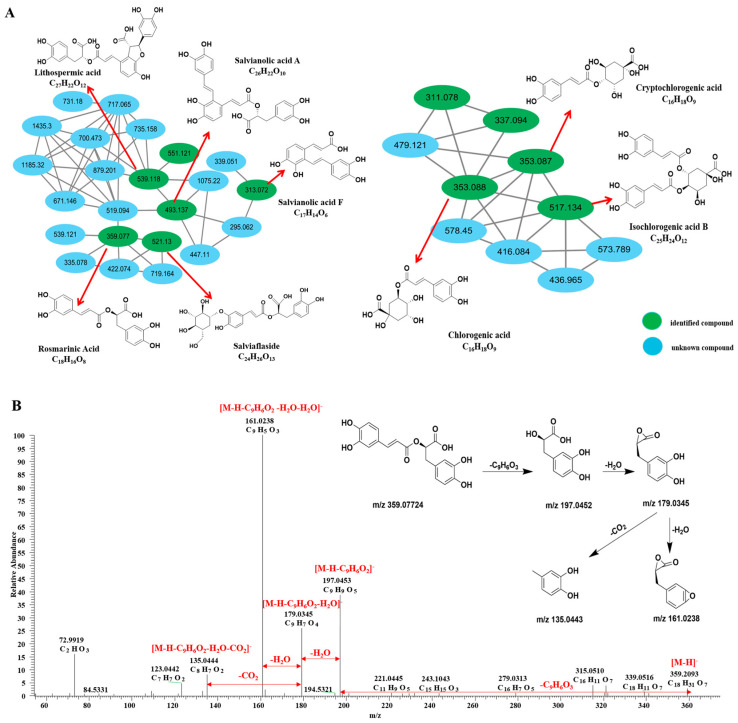
Phenolic acid structure based on MS^2^ spectral analysis. (**A**) MN of phenolic acid compounds based on their characteristics. (**B**) The MS/MS fragmentation pathways of rosmarinic acid in the extract of XBKF capsules.

Ten nodes are interconnected, forming a cluster (Figure 2A). Among these nodes, cryptochlorogenic acid (39, *m*/*z* 353.0878), chlorogenic acid (46, *m*/*z* 353.0878), coumaroyl quinic acid (57, *m*/*z* 337.09289), and isochlorogenic acid B (80, *m*/*z* 517.13405) can be annotated using GNPS. Peak 28 displays excimer ions [M-H]^−^ at *m*/*z* 169.01424 (C_7_H_5_O_5_^−^), with sub-ions at *m*/*z* 125.0235 resulting from the loss of CO_2_. According to the PubChem databases, as well as the data reported by Wang et al. (2015) [23], peak 28 is identified as gallic acid.

Peak 36 appears at 2.59 min, where the parent ion [M-H]^−^ undergoes cleavage at *m*/*z* 153.01933, producing daughter ions at *m*/*z* 109.0285 [M-H-CO_2_]^−^ and 108.0207 [M-H-CO_2_-H]^−^. Integrating data from the data reported by Lv et al. (2023) [26], peak 36 is identified as gentisic acid. Peak 38 in the *m*/*z* 181.04953 (C_9_H_9_O_4_^+^) position shows an excimer ion [M + H]^+^, through the continuous loss of H_2_O [M + H-H_2_O]^+^ fragment ions, *m*/*z* 163.0383 (C_9_H_7_O_3_^+^) and [M + H-2H_2_O]^+^ fragment ions, and *m*/*z* 145.0278 (C_9_H_5_O_2_^+^). Further neutral loss of CO produces *m*/*z* 135.0435 [M + H-C_8_H_7_O_2_]^+^. Based on data reported by Shi et al. (2025) [27], along with the standard, peak 38 was accurately identified as caffeic acid.

Peak 39, associated with the presence of the caffeoyl group at the C4 hydroxyl position of quinic acid, exhibits a steric hindrance effect that favors its cleavage, leading to the direct formation of caffeic acid fragments with *m*/*z* 179.0345 (C_9_H_7_O_4_^−^), alongside the neutral loss of dehydrated quinic acid (C_7_H_10_O_5_). Additionally, the secondary fragment at *m*/*z* 135.0443 [C_9_H_8_O_4_-CO_2_-H]^−^ arises from the decarboxylation of *m*/*z* 179.0345. Although the cleavage pathways for these fragments are fundamentally similar, variations in the positions of the acyl groups result in slight differences in the relative intensities of the fragment ions, which can be distinguished by their retention times. Consequently, utilizing GNPS, OTCML, and the chromatographic data, along with the fragmentation patterns from the standard, peak 39 was accurately identified as cryptochlorogenic acid.

The excimer ion peak at *m*/*z* 353.08780 (C_16_H_17_O_9_, [M-H]^−^) is observed in the primary mass spectrometry of compound **46**. In the secondary mass spectrometry, the fragment ion peak corresponding to the quinic acid fragment is noted at *m*/*z* 191.0558 (C_7_H_11_O_6_^−^). Additionally, the fragment ion associated with the caffeic acid group appears at *m*/*z* 179.0344 (C_9_H_7_O_4_^−^). This ion subsequently loses one molecule of CO_2_, resulting in the decarbonylation fragment ion of the caffeic acid group at *m*/*z* 135.0443 (C_8_H_7_O_2_^−^). Alternatively, the loss of one molecule of H_2_O leads to the formation of *m*/*z* 161.0237 (C_9_H_5_O_3_^−^). It is inferred that compound **46** is chlorogenic acid.

Compounds **48** and **66** both displayed excimer ions [M-H]^−^ at *m*/*z* 193.05063 (C_10_H_9_O_4_^−^). Compound **48** loses a neutral CO_2_ molecule, forming the [M-H-CO_2_]^−^ ion at *m*/*z* 149.0601 (C_9_H_9_O_2_^−^). This process occurs through a rearrangement reaction involving the adjacent phenolic hydroxyl group. The C-O bond in the methoxy group undergoes homolytic cleavage, releasing a neutral methyl radical (-CH_3_) and generating a stable orthoquinone radical anion at *m*/*z* 178.0266 [M-H-CH_3_]^−^. Subsequently, it loses CO_2_ to yield the ion at *m*/*z* 134.0365 [M-H-CH_3_-CO_2_]^−^. Due to the proximity of the hydroxyl and methoxy groups in ferulic acid, this rearrangement reaction proceeds with relative ease. Hence, the ion *m*/*z* 178.0266 (C_9_H_6_O_4_^−^) typically exhibits a high abundance in its MS/MS spectrum. Conversely, ethyl caffeate, hindered by the positioning of its hydroxyl and methoxy groups, cannot easily undergo a favorable cyclic transition state, thereby impeding the CH_3_ loss pathway. In compound **66**, the cleavage is influenced by the structure of the substituent at the central position. The ion *m*/*z* 149.0238 [M-H-CO_2_]^−^ is generated through a direct decarboxylation reaction, with the abundance of *m*/*z* 178.0269 (C_9_H_6_O_4_^−^) being relatively lower. The sole discrepancy lies in the ion peak response value. Upon comparison with OTCML and data reported by Shi et al. (2025) [27] and standards, ferulic acid was confirmed as compound **48**, while isoferulic acid was identified as compound **66**.

Peak 76 exhibits the excimer ion [M-H]^−^ at *m*/*z* 521.13006 (C_24_H_25_O_13_^−^). The glycosidic moiety is lost as a neutral molecule, C_15_H_18_O_9_ (342.0952 Da), resulting in the formation of *m*/*z* 179.0345 (C_9_H_7_O_4_^−^). This ion subsequently undergoes decarboxylation, yielding *m*/*z* 135.0443 (C_8_H_7_O_2_^−^). Peak 76 has been identified as salviaflaside.

Peak 88 displays the precursor ion peak [M + H]^+^ at *m*/*z* 209.08083 (C_11_H_13_O_4_^+^), with the subsequent loss of a neutral molecule C_8_H_10_O_3_ in the secondary fragmentation, yielding *m*/*z* 55.0184 (C_3_H_3_O^+^). Simultaneously, the loss of one molecule of H_2_O results in the formation of a fragment ion peak at *m*/*z* 191.0696 (C_11_H_11_O_3_^+^). Through comparison with the OTCML, compound **88** was conclusively identified as ethyl caffeate.

Peaks 80, 84, and 94, in positive-ion mode, all exhibit excimer ions [M + H]^+^ at *m*/*z* 517.13405 (C_25_H_25_O_12_^+^). In the secondary mass spectrometry, characteristic fragments are generated by the continuous loss of caffeyol groups through the cleavage of acyl–oxygen bonds. A distinct fragment ion peak *m*/*z* 163 (C_9_H_7_O_3_^+^) emerged. Further, one molecule of H_2_O was lost to form *m*/*z* 145 (C_9_H_5_O_2_^+^), and one molecule of CO was lost to generate *m*/*z* 117 (C_8_H_5_O^+^). It is speculated that its molecular formula is C_25_H_24_O_12_. Based on the signal strength of the elution and the elution sequence in reversed-phase chromatography, and given the comparison with the OTCML databases, GNPS isochlorogenic acid B, isochlorogenic acid A, and isochlorogenic acid C, the compounds were finally identified as isochlorogenic acid B, isochlorogenic acid A, and isochlorogenic acid C, respectively.

Peak 93, at *m*/*z* 359.07724 (C_18_H_15_O_8_^−^), shows the excimer ion [M-H]^−^ through the loss of the coffee acyl group 179.0339. The characteristic fragment ions at *m*/*z* 161.0238 [M-H-C_9_H_6_O_3_]^−^ and 197.0452 [M-H-C_9_H_6_O_3_-2H_2_O]^−^ were generated. Therefore, based on the data reported by Liu et al. (2024) [28], the MS/MS fragmentation pathways of rosmarinic acid are shown in Figure 2B. It has been identified as rosmarinic acid.

#### 2.1.3. Identification of Terpenoids

Peak 129 exhibits an excimer ion peak [M + H]^+^ at *m*/*z* 469.33123 (C_27_H_45_O_3_^+^). The neutral fragment [M + H-C_8_H_16_O_2_]^+^ results in *m*/*z* 273.2208 (C_19_H_29_O). Subsequent loss of H_2_O produces *m*/*z* 255.2103. Rearrangement via RDA cleavage generates *m*/*z* 161.1323 and *m*/*z* 69.0704. Cleavage of the parent ion [M + H]^+^ yields isoprene fragment ions [C_5_H]^+^. Comparison with the database and GNPS confirms that component **129** is Sarsasapogenin.

**Figure 3:**
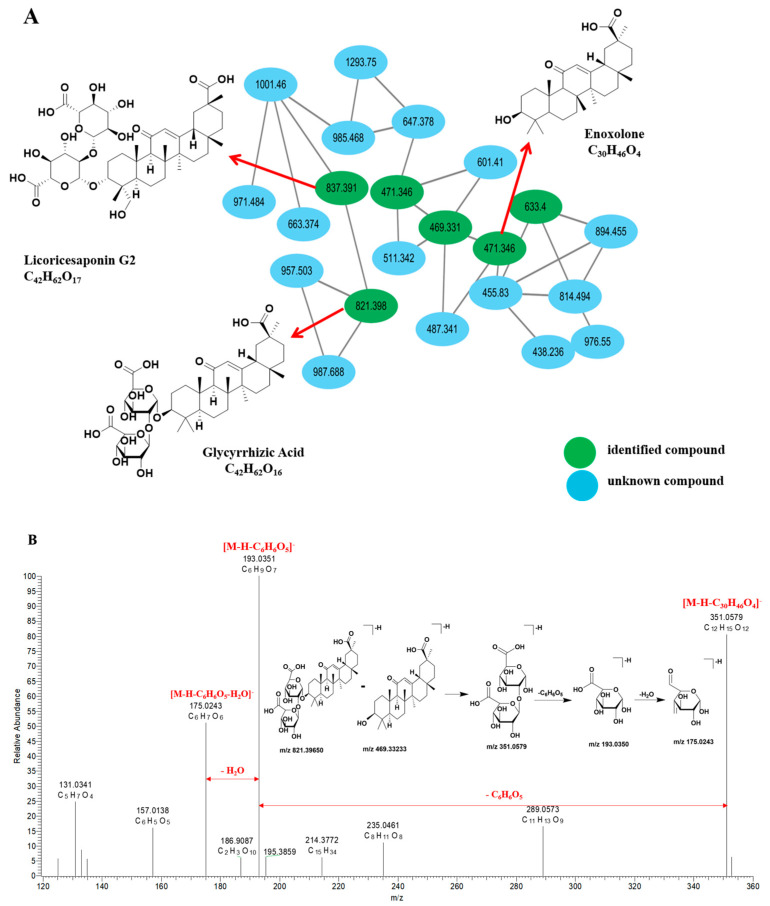
Terpene structure based on MS^2^ spectral analysis. (**A**) MN of terpene compounds based on their characteristics. (**B**) The MS/MS fragmentation pathways of glycyrrhizic acid in the extract of XBKF capsules.

Using peak 135 (t_R_ = 17.63 min) as a case study for identification and analysis, the excimer ion peak [M-H]^−^ for this component was observed at *m*/*z* 821.39650 (C_42_H_61_O_16_^−^). Subsequent secondary cleavage produced a fragment ion peak at *m*/*z* 351.0579 [M-H-C_30_H_46_O_4_]^−^, followed by the loss of C_6_H_6_O_5_, resulting in a fragment ion peak at *m*/*z* 193.0350 (C_6_H_9_O_7_^−^). Ultimately, the loss of one H_2_O molecule yielded a fragment ion peak at *m*/*z* 175.0243 (C_6_H_7_O_6_^−^). The fragmentation pathways are illustrated in Figure 3B. Additionally, by comparing these findings with the relevant data reported by Pan et al. (2025) [22] and standard substances, it was concluded that component **135** is glycyrrhizic acid.

Peak 136 (t_R_ = 17.65 min) corresponds to the excimer ion peak [M + H]^+^ with an *m*/*z* of 471.34688 (C_30_H_47_O_4_^+^). Subsequent secondary cleavage, resulting from the protonation of its hydroxyl or carboxyl groups, leads to the loss of one molecule of water, generating a fragment ion peak at *m*/*z* 453.3346 (C_30_H_45_O_3_^+^). The continuous loss of a neutral carbon monoxide molecule [M + H-H_2_O-CO]^+^ results in an ion at *m*/*z* 425.3401 (C_29_H_45_O_2_^+^). Through RDA cleavage, an ion at *m*/*z* 317.21042 (C_20_H_29_O_3_^+^) is produced, followed by the loss of C_4_H_6_ (54.0469 Da), yielding *m*/*z* 263.1634 (C_16_H_23_O_3_^+^). Further loss of CO (27.9949 Da) results in an ion at *m*/*z* 235.1687 (C_15_H_23_O_2_^+^). Comparison with relevant data reported by Pan et al. (2025) [22] and GNPS analysis identified component **136** as enoxolone.

#### 2.1.4. Identification of Quinone Compounds

Peak 115 eluted at 13.58 min. Under positive-ion detection, the ion *m*/*z* 285.07575 [M + H]^+^ was observed, with the loss of one methanol molecule [M + H-CH_3_OH]^+^. Fragment ion *m*/*z* 253.0497 (C_15_H_9_O_4_^+^), lacking a methyl group, yielded *m*/*z* 270.0524 (C_15_H_10_O_5_^+^). Subsequent elimination of a COOH radical (45 Da) resulted in *m*/*z* 225.0548 (C_14_H_9_O_3_^+^). Through comparison with appropriate standards and the data reported by Zeng et al. (2024) [29], the compound was identified as physcion.

Using emodin as a case study, a peak at 118 in the positive-ion mode yields an excimer ion peak at *m*/*z* 271.06009 [M + H]^+^. The presence of one hydroxyl (-OH) group and one methyl (-CH_3_) group on either the A or C ring of the emodin molecule results in the loss of one neutral C_2_H_2_O molecule (42.0106 Da), leading to the formation of *m*/*z* 229.0499 [M + H-C_2_H_2_O]^+^. Additionally, the removal of one carbon monoxide molecule (CO, −28 Da) generates *m*/*z* 201.05498. The elimination of another CO molecule results in *m*/*z* 243.0652 [M + H-CO]^+^. By integrating data from OTCML and relevant standards, it was confirmed that this compound is emodin, and the lysis rule is illustrated as described.

Using peak 163 as a case in point, this compound exhibits a strong response in positive-ion mode. It was identified as cryptotanshinone based on data reported by Shen et al. (2022) [30]. The primary mass spectrometer generates an excimer ion peak at *m*/*z* 297.14852 [M + H]^+^ with a retention time of 21.89 min. The secondary mass spectrometer produces fragment ions at *m*/*z* 279.1368 and 251.1419. The ion at 237.0904 corresponds to [M + H-H_2_O]^+^, [M + H-H_2_O-CO]^+^, and [M + H-H_2_O-CO-2CH_3_]^+^.

The excimer ion peak 148 at *m*/*z* 311.12778 [M + H]^+^ can lose water molecules sequentially to produce *m*/*z* 293.11636 (C_19_H_17_O_3_^+^) and *m*/*z* 275.1055 (C_19_H_15_O_2_^+^). Subsequent elimination of ethylene [M + H-C_2_H_4_] results in *m*/*z* 283.1319 (C_18_H_19_O_3_^+^), followed by the loss of H_2_O to yield *m*/*z* 265.1213 (C_18_H_17_O_2_^+^). Comparison of secondary mass spectrometry fragments with databases, the data reported by Sun et al. (2019) [31], and standard substances confirmed that peak 148 corresponds to tanshinone IIB. The excimer ion peak 168 at *m*/*z* 295.13280 [M + H]^+^ corresponds to peak 168. During the cleavage process, one molecule of H_2_O is readily eliminated, followed by the continuous removal of CO, resulting in the formation of *m*/*z* 277.1213 (C_19_H_17_O_2_^+^) and *m*/*z* 249.1263 (C_18_H_17_O^+^). The loss of one methyl molecule, represented as [M + H-H_2_O-CH_3_]+, yields *m*/*z* 262.0979 (C_18_H_14_O_2_^+^), while the elimination of a neutral methyl fragment produces *m*/*z* 280.1087 [M + H-CH_3_]^+^. Based on secondary mass spectrometry fragments obtained from the PubChem databases, along with the data reported by Liu et al. (2024) [28], tanshinone IIA was identified.

#### 2.1.5. Identification of Coumarin Compounds

Peak 47, with an *m*/*z* of 177.0193 [M-H]^−^ in negative ion detection mode, underwent CO_2_ and CO loss, forming fragment ions at *m*/*z* 133.0286 [M-H-CO_2_]^−^, *m*/*z* 149.0237 [M-H-CO]^−^, and *m*/*z* 105.0335 [M-H-3CO]^−^. Compound **47** was identified as daphnetin through comparison with reports in the data reported by Pan et al. (2025) [22]. Peak 56 had a retention time of 7.27 min, with an *m*/*z* of 193.04953 [M + H]^+^ in positive ion detection mode. In secondary mass spectrometry, excimer ions lost methyl radicals or CH_3_OH, resulting in *m*/*z* 178.02557 [M + H-CH_3_]^+^ and *m*/*z* 161.0591 [M + H-CH_3_OH]^+^, followed by the sequential loss of two CO molecules to generate fragment ions at *m*/*z* 133.0643 [M + H-CH_3_OH-CO], *m*/*z* 165.0540 [M + H-CO]^+^, and *m*/*z* 137.0591 [M + H-2CO]. Direct loss of one molecule of methanol [M + H-CH_3_OH]^+^ yielded *m*/*z* 134.0596 (C_8_H_6_O_2_^+^). Compound **56** was identified as scopoletin through comparison with reports in the data reported by Yang et al. (2025) [19].

Peak 92 exhibited a mass-to-charge ratio (*m*/*z*) of 161.02441 [M-H]^−^ in negative-ion detection mode and subsequently lost one molecule of neutral carbon monoxide (CO), resulting in the formation of *m*/*z* 133.0287 (C_8_H_5_O_2_^−^). A comparison with the data reported by Zhu et al. (2022) [25] led to the identification of compound **92** as 7-hydroxycoumarin. The retention time for peak 120 was recorded at 14.29 min. In positive ion detection mode, *m*/*z* 315.04992 [M + H]^+^ was observed, indicating the cleavage of the lactone ring (C ring). The loss of one molecule of neutral CO produced fragment ions at *m*/*z* 287.05590 [M + H-CO]^+^ and 259.0607 [M + H-2CO]^+^, while the loss of one molecule of neutral water (H_2_O) resulted in fragment ions at *m*/*z* 297.0401 [M + H-H_2_O]^+^. Based on comparisons with the PubChem databases and data reported by Lee et al. (2010) [32], compound **120** was identified as wedelolactone.

## 3. Discussion

This study uses UHPLC-Q-Exactive Orbitrap MS combined with MN technology for rapid qualitative identification of the characterizations in XBKF capsules, demonstrating the unparalleled synergy between the high-resolution power of the UHPLC-Q-Exactive Orbitrap and the high-throughput classification of MN. This approach overcomes the high-cost and low-efficiency issues associated with traditional methods used to discover constituents in TCM [33].

Pharmacological studies have shown that the seven plants in XBKF capsules have definite pharmacological activities, including antioxidant, anti-inflammatory, and promoting blood circulation and removing blood stasis and freckles effects. *Salvia miltiorrhiza Bunge* contains cryptotanshinone, salvianolic acid B, and rosmarinic acid (Peak 79, Rt 10.71 min). As a phenolic acid compound, rosmarinic acid can protect cells from oxidative damage, with potential applications in the treatment of conditions related to inflammation and oxidative stress [34]. *Fructus mori* has been reported to be rich in flavonoids, polyphenols, alkaloids, and anthocyanins, which have the effects of anti-oxidation, anti-inflammation, and melanin synthesis-inhibiting [35]. *Tribulus terrestris* L. is rich in flavonoids (quercetin) and alkaloids, which have significant effects on promoting blood circulation and removing blood stasis [36]. *Typhonium giganteum* extracts have anti-inflammatory effects via regulation of NF-κB signaling and ROS production, which contribute to ameliorating dermatological inflammation and hyperpigmentation disorders [37]. The pharmacological activities of *Glycyrrhiza uralensis,* including the antiviral and antimicrobial activities, have been most commonly reported, such as liquiritigenin [38]. Additionally, *Eclipta prostrata* and *Fallopia multiflora* contain coumarin derivatives, flavonoids, and triterpene saponins, exhibiting antibacterial, anticancer, hepatoprotective, and hair growth-promoting effects [39].

In this study, the identification of compounds was based on the relevant literature, databases, and multiple verifications by retention time and fragment ions. Consequently, it is logical to think that it comes from one of the components of the sample. It is worth mentioning that XBKF capsules are a prescription comprising seven herbs. Each herb is recognized for containing a significant number of compounds, and some compounds may appear in multiple herbs [40]. If a compound is identified that has not been previously described in any of the seven plants, it may be the result of complex chemical changes that have occurred gradually during the process of TCM processing. Moreover, newly formed chemical constituents may be the basis of clinical efficacy [41]. Another plausible explanation is that some compounds may have always existed in the individual herbs but remained undetected due to technological limitations of earlier studies, such as low sensitivity and being incapable of distinguishing compounds with very similar molecular mass [42]. With the advanced sensitivity and resolution of modern UHPLC-Orbitrap MS technology, it is now possible to detect trace constituents that were previously unrecognized. For example, Włodarczyk et al. (2022) [43] identified approximately 90 previously unreported saponins in *Strophanthus* seeds using UHPLC-MS/MS. Therefore, this study demonstrates an efficient method to identify compounds in complex herbal drug mixtures using UHPLC-Orbitrap MS and MN tools, which will support future research into quality controls and treatment mechanisms of TCM preparations.

By automatically searching the vast GNPS database and analyzing the generated MN, we can get more information for identification. The resulting molecular clusters may indicate potential compound types present in TCM. However, there are two significant concerns. There is a possibility of false positives in molecular network clustering analysis based on the similarity of MS/MS spectrometry in GNPS, such as molecules with clustering structures not necessarily sharing similar MS secondary spectra. In addition, the annotation efficiency of molecular network clusters is not high; compounds in some clusters cannot be identified [44]. After drawing a comparison with other reference standards and screening out the unreasonable redundant nodes and wrong annotations, 34 constituents were finally obtained from the MN [45]. Consequently, this research not only provides a solid platform for the further development of XBKF capsules but also demonstrates a versatile analytical workflow with broad applicability to other traditional Chinese medicine prescriptions.

## 4. Materials and Methods

### 4.1. Materials and Reagents

XBKF capsules were obtained from Hunan Medical College General Hospital (Huaihua, Hunan, China; batch number: 20240829). Chromatographic-grade methanol and acetonitrile were procured from Merck (Kenilworth, NJ, USA). Distilled water was sourced from Watsons Food & Beverage Co., Ltd. (Guangzhou, China). LC-MS grade formic acid was acquired from Fisher Scientific (Waltham, MA, USA). All other reagents used were of analytical grade. A total of 22 reference standards were utilized and details of these 22 reference standards are provided in Table S2.

### 4.2. Sample Preparation

The contents of the XBKF capsules (0.5 g) were subjected to ultrasonic extraction using 5 mL of 70% methanol at room temperature for 30 min. The mixture was then centrifuged at 13,523× *g* for 20 min and filtered through a 0.22 μm microporous filter prior to LC-MS analysis. The 22 standards were dissolved in methanol at a concentration of approximately 1 mg/mL. Each stock solution was then further diluted via mixing to obtain a standard mixture (approximately 30 μg/mL) [25]. Subsequently, the mixture was centrifuged at 13,523× *g* for 20 min, and these solutions were stored at 4 °C for analysis.

### 4.3. Liquid Chromatographic Conditions

To achieve improved chromatographic peak shape and separation resolution, several parameters were established during the detection and identification process. These included the chromatographic column (Thermo Syncronis aQ C18 (Thermo Fisher Scientific, San Jose, CA, USA), 2.1 × 100 mm, 1.7 µm), the column temperature (40 °C), and the mobile phase gradient. Each LC-MS analysis was interfaced with the Thermo Scientific Dionex Ultimate 3000 RS via an ESI source (Thermo Fisher Scientific Co., Ltd., Waltham, MA, USA) and performed using the Q-Exactive Focus Orbitrap MS. The mobile phase consisted of a 0.1% formic acid aqueous solution (C) and acetonitrile (D). The flow rate was set at 0.28 mL/min, and the optimized gradient elution program was as follows: from 0 to 5 min, 5–15% C; from 5 to 10 min, 15–30% C; from 10 to 15 min, 30–50% C; from 15 to 20 min, 50–75% C; from 20 to 28 min, 75–95% C; and from 28 to 33 min, 95–5% C, with an injection volume of 2 µL.

### 4.4. MS Spectrometry Conditions

Mass spectrometry analysis was conducted using the Q-Exactive Orbitrap MS (Thermo Fisher Scientific, Bremen, Germany), which is equipped with a heated electrospray ionization source (HESI). Data acquisition in both positive- and negative-ion modes was performed via full-scan data-dependent MS/MS (Full-scan-DDMS^2^), covering a mass range of *m*/*z* 100–1500. The mass spectrometry parameters were established as follows: the capillary voltage was set to 3.5 kV for positive-ion mode and 3.0 kV for negative-ion mode. The full mass resolution was configured to 70,000. The heater temperature was maintained at 350 °C, while the heated capillary tube temperature was set to 320 °C. The flow rates for sheath gas and auxiliary gas were 30 and 10 arb, respectively. The radio frequency (RF) level of the S-lens was set at 50. The resolution for ddMS^2^ was established at 17,500. Fragmentation was performed using step-normalized collision energies (NCEs) of 35%. Data collection and analysis were conducted using Xcalibur 4.2 software (Thermo Fisher Scientific, San Jose, CA, USA).

### 4.5. Integrated Strategy for Data Analysis

The purpose of this study is to systematically identify the chemical components of XBKF. An effective strategy has been established to comprehensively and accurately characterize the chemical composition of XBKF. The strategy comprises four main steps.

First, the chemical compounds in XBKF capsules were extracted and enriched using a 70% methanol ultrasonic extraction method. To achieve good separation and an abundant signal response, the proportion and variety of mobile phases, including acetonitrile-aqueous, methanolaqueous, acetonitrile-aqueous with 0.1% formic acid, and methanolaqueous with 0.1% formic acid, were optimized to obtain better chromatographic conditions. Thus, the mobile phases consisted of acetonitrile-aqueous with 0.1% formic acid, which could be considered as the most optimized separation condition; the proportion used is outlined in Section 4.3. Secondly, data from PubMed (https://pubmed.ncbi.nlm.nih.gov/, accessed on 6 June 2025), PubChem (https://pubchem.ncbi.nlm.nih.gov/, accessed on 6 June 2025), Web of Science (https://www.webofknowledge.com, accessed on 6 June 2025), CNKI (https://www.cnki.net, accessed on 6 June 2025), Google Scholar, and Wanfang Data Knowledge Service Platform were used to summarize the mass spectrometry information of XBKF-related components and identify the target components. The retrieved data encompass component names, relative molecular masses, ion modes, parent ion *m*/*z* values, and MS/MS spectral fragments to create a compound database. Unknown compounds are identified by comparing quasi-molecular ions and MS^2^ fragment ions against the internal library. Thirdly, GNPS was used to detect and identify unknown compounds based on fragment similarity. The original MS/MS spectral data were converted into the mzML format that contains all the analytical information. Then, the mzML format file was uploaded to the client through the FileZilla software (3.58.0) and imported into the GNPS platform (http://gnps.ucsd.edu, accessed on 6 June 2025) for analysis. Furthermore, the MS^2^ fragment ions were visualized on the GNPS platform within Cytoscape 3.10.4 to expedite precise analysis and modification of the complete molecular network dataset. Finally. Xcalibur 4.4 software was used to extract and match the peaks of the XBKF mass spectrum data. The relative molecular mass of the parent ion was calculated according to the information of the excimer ion or the additive ion, and its molecular formula was deduced. To satisfy a mass deviation of less than 5, the chemical composition of XBKF was identified and analyzed according to the fragment ion information provided by the GNPS platform and database and combined with the fragmentation law of the reference substance and compound mass spectrum.

### 4.6. Molecular Network Analysis Based on GNPS

Using the MsConvert software (3.0.19147), LC-MS/MS raw files (raw) were converted into mzXML format files, which were subsequently uploaded to the GNPS platform (https://gnps.ucsd.edu, accessed on 6 June 2025) via the FileZilla client to create molecular networks. The network parameters were configured with a Precursor Ion Mass Tolerance of 2.0 Da and a Fragment Ion Mass Tolerance of 0.5 Da. Additionally, the cosine fraction threshold (Min PairsCos) was set to 0.7, the Minimum Matched Fragment Ions to 6, and the Network TopK was set to 10. All the remaining parameters were maintained at their default settings. The final molecular network was visualized and analyzed using Cytoscape software (3.9.1).

## 5. Conclusions

In this study, we established a convenient and reliable analytical strategy combining UHPLC-Q-Exactive Orbitrap MS with GNPS-based MN to comprehensively characterize XBKF capsules. A total of 170 chemical components were successfully analyzed from XBKF capsules were successfully analyzed. These components encompass flavonoids, phenolic acids, quinones, alkaloids, terpenoids, phenylpropanes, and other substances. Moreover, numerous unassigned clusters and nodes were observed in the GNPS, which is conducive to the discovery of novel compounds. Future studies should focus on using more computational tools to discover compounds and developing automated approaches to investigate complex TCM prescriptions. The results of this study lay the foundation for in-depth research on the quality control of XBKFcapsules and promote the development of modern XBKF capsules prescription.

## Figures and Tables

**Table 1:** Identification of chemical components in XBKF by UHPLC-Q-Exactive Orbitrap MS.

Peak	t_R_ (min)	Theoretical Mass *m*/*z*	Experimental Mass *m*/*z*	Error (ppm)	Formula	Identification	Peak	t_R_ (min)	Theoretical Mass *m*/*z*	Experimental Mass *m*/*z*	Error (ppm)	Formula	Identification
1	0.69	259.02244	259.02286	1.615	C_6_H_13_O_9_P	Glucose-6-Phosphate	86 *	10.37	285.04046	285.04092	1.609	C_15_H_10_O_6_	Luteolin
2	0.75	665.21458	665.21600	2.133	C_24_H_42_O_21_	Stachyose	87	10.47	433.11402	433.11472	1.616	C_21_H_22_O_10_	Naringenin-7-O-glucoside
3	0.78	387.11441	387.11526	2.031	C_13_H_24_O_13_	2,3,4,5,6-pentahydroxy-7-[(2S,3R,4S,5S,6R)-3,4,5-trihydroxy-6-(hydroxymethyl)oxan-2-yl]oxyheptanoic acid	88	10.52	209.08083	209.08026	−2.752	C_11_H_12_O_4_	Ethyl caffeate
4 *	0.79	341.10893	341.10950	1.657	C_12_H_22_O_11_	Sucrose	89	10.60	301.07066	301.06964	−3.403	C_16_H_12_O_6_	Fallacinol
5 *	0.80	179.05611	179.05568	−2.409	C_6_H_12_O_6_	D-Galactose	90	10.65	1079.52687	1079.52441	−2.288	C_51_H_82_O_24_	Terrestrosin K
6	0.81	191.05611	191.05576	−1.839	C_7_H_12_O_6_	Quinic acid	91	10.67	654.36953	654.36780	−2.646	C_30_H_55_NO_14_	Morusimic acid C isomer + Glu
7 *	0.82	149.04554	149.04485	−6.074	C_5_H_10_O_5_	D-ribose	92	10.71	161.02441	161.02371	−4.393	C_9_H_6_O_3_	7-Hydroxycoumarin
8	0.82	195.05102	195.05072	−1.722	C_6_H_12_O_7_	Gluconic acid	93 *	10.71	359.07724	359.07797	2.031	C_18_H_16_O_8_	Rosmarinic Acid
9	0.85	177.04046	177.04001	−2.549	C_6_H_10_O_6_	Gluconolactone	94	10.87	517.13405	517.13257	−2.867	C_25_H_24_O_12_	Isochlorogenic acid C
10	0.86	209.03029	209.03012	−0.816	C_6_H_10_O_8_	Mucic acid	95	10.98	537.10384	537.10492	1.994	C_27_H_22_O_12_	Salvianolic acid H/J
11	0.87	266.12342	266.12234	−4.07	C_10_H_19_NO_7_	Fructose-Ethylglycine	96	10.98	493.11402	493.11508	2.15	C_26_H_22_O_10_	Salvianolic acid A
12	0.87	161.04554	161.04495	−3.705	C_6_H_10_O_5_	3-Hydroxy-3-methylglutaric acid	97	11.02	341.06557	341.06454	−3.047	C_18_H_12_O_7_	Salvianolic acid G
13 *	0.88	133.01424	133.01343	−6.289	C_4_H_6_O_5_	Malic acid	98 *	11.38	329.10306	329.10361	1.667	C_18_H_18_O_6_	Acetylshikonin
14	0.88	278.12342	278.12238	−3.77	C_11_H_19_NO_7_	Fructose-Proline	99	11.68	551.17591	551.17450	2.531	C_26_H_30_O_13_	Liquiritin apioside
15	0.88	138.05495	138.05449	−3.369	C_7_H_7_NO_2_	2-Aminobenzoic acid	100	11.75	139.03897	137.02286	−3.361	C_7_H_6_O_3_	Protocatechualdehyde
16	0.88	290.08813	290.08862	1.656	C_11_H_17_NO_8_	N-Fructosyl pyroglutamate	101	11.76	717.14610	717.14752	1.885	C_36_H_30_O_16_	Salvianolic acid B
17	0.91	173.00916	173.00864	−3.012	C_6_H_6_O_6_	Cis-Aconitic acid	102	12.10	419.13365	419.13248	2.812	C_21_H_22_O_9_	Isoliquiritin
18 *	0.91	191.01972	191.01933	−2.072	C_6_H_8_O_7_	Citric acid	103	12.48	551.11949	551.12085	3.466	C_28_H_24_O_12_	Salvianolic acid isomers
19	0.93	130.08625	130.08589	−2.807	C_6_H_11_NO_2_	Pipecolic acid	104 *	12.64	255.06628	255.06676	1.874	C_15_H_12_O_4_	Liquiritigenin
20	1.18	124.0393	124.03903	−2.217	C_6_H_5_NO_2_	Nicotinic acid	105	12.64	285.07684	285.07751	2.326	C_16_H_14_O_5_	Licochalcone B
21	1.18	290.08813	290.08871	1.966	C_11_H_17_NO_8_	Fructose-Pyrrolidonecarboxylic acid	106	12.74	253.05063	253.05112	1.928	C_15_H_10_O_4_	Daidzein
22	1.23	344.13399	344.13266	−3.374	C_15_H_21_NO_8_	Fructose-tyrosine	107	12.80	201.11323	201.11310	−0.685	C_10_H_18_O_4_	3-tert-Butyladipic acid
23	1.23	182.08116	182.08061	−3.074	C_9_H_11_NO_3_	L-Tyrosine	108	12.87	285.04046	285.04120	2.592	C_15_H_10_O_6_	Citreorosein
24	1.25	147.02989	147.02925	−4.398	C_5_H_8_O_5_	α-Hydroxyglutaric acidα	109	12.90	373.09289	373.09366	2.062	C_19_H_18_O_8_	Methyl rosmarinate
25	1.29	117.01933	117.01845	−7.537	C_4_H_6_O_4_	Succinic Acid	110	13.05	299.05611	299.05676	2.169	C_16_H_12_O_6_	Hispidulin
26 *	1.33	152.05668	152.05615	−3.528	C_5_H_5_N_5_O	Guanine	111	13.11	551.11949	551.12122	3.112	C_28_H_24_O_12_	Paederosidic acid methyl ester
27	1.38	292.14017	292.14087	2.378	C_12_H_23_NO_7_	Fructose-L-isoleucine	112	13.15	556.21772	556.21808	0.643	C_29_H_33_NO_10_	Isoflavone base + 1O, 1MeO, O-Hex + C_7_H_12_NO
28	1.73	169.01424	169.01373	−3.056	C_7_H_6_O_5_	Gallic acid	113	13.37	339.10744	339.10745	0.018	C_16_H_18_O_8_	Gerberinside
29	2.19	328.13907	328.13770	−4.201	C_15_H_21_NO_7_	Fructose-phenylalanine	114	13.48	431.13365	431.13159	−4.789	C_22_H_22_O_9_	Ononin
30	2.19	329.08780	329.08859	2.384	C_14_H_18_O_9_	Phenylacetic acid + 2O, O-Hex	115 *	13.58	285.07575	285.07587	0.421	C_16_H_12_O_5_	Physcion
31	2.27	166.08625	166.08572	−3.222	C_9_H_11_NO_2_	L-Phenylalanine	116	13.58	285.07575	285.07580	0.421	C_16_H_12_O_5_	Wogonin
32	2.53	220.11794	220.11717	−3.540	C_9_H_17_NO_5_	Pantothenic acid	117	13.61	441.20201	441.20233	0.718	C_24_H_28_N_2_O_6_	Diferuloyl putrescine
33	2.54	218.10339	218.10336	−0.165	C_9_H_17_NO_5_	D-pantothenic acid	118 *	13.91	271.06009	271.06042	1.181	C_15_H_10_O_5_	Emodin
34	2.56	197.04554	197.04530	−1.252	C_9_H_10_O_5_	Salvianic acid A	119	13.92	307.07243	307.07303	1.954	C_17_H_12_N_2_O_4_	Flazin
35	2.58	417.08271	417.08142	−3.117	C_20_H_18_O_10_	Salvianolic acid D	120	14.29	315.04992	315.05057	2.034	C_16_H_10_O_7_	Wedelolactone
36	2.59	153.01933	153.01868	−0.652	C_7_H_6_O_4_	Gentisic acid	121	14.45	461.10893	461.11002	2.354	C_22_H_22_O_11_	Peonidin-3-O-beta-galactoside
37	3.31	153.01933	153.01868	−4.261	C_7_H_6_O_4_	Protocatechuic acid	122	14.93	271.06119	271.06189	2.558	C_15_H_12_O_5_	Naringenin
38 *	4.31	181.04953	181.04884	−3.84	C_9_H_8_O_4_	Caffeic acid	123	15.04	433.33123	433.33319	4.508	C_27_H_44_O_4_	Gitogenin
39 *	4.32	353.08780	353.08853	2.052	C_16_H_18_O_9_	Cryptochlorogenic acid	124	15.61	271.09648	271.09741	3.410	C_16_H_14_O_4_	Retrochalcone
40 *	4.91	137.02441	137.02371	−5.162	C_7_H_6_O_3_	4-hydroxybenzoic acid	125	15.66	875.41044	875.41248	2.325	C_42_H_68_O_17_S	Eclalbasaponin VI
41	5.07	175.06119	175.06073	−2.666	C_7_H_12_O_5_	2-Isopropylmalic acid	126	15.93	445.11402	445.11496	2.112	C_22_H_22_O_10_	Calycosin-7-O-β-D-glucoside
42	5.07	165.05571	165.05516	−3.377	C_9_H_10_O_3_	2-phenoxypropanoicacid	127	15.97	313.07176	313.07248	2.295	C_17_H_14_O_6_	Salvianolic acid F
43	5.54	577.13514	577.13696	3.137	C_15_H_11_O_6_	Cyanidin isomer	128	16.16	469.33123	469.33209	1.819	C_30_H_44_O_4_	Glabrolide
44 *	5.54	577.13514	577.13696	1.811	C_30_H_26_O_12_	Procyanidin B1	129	16.82	417.33632	417.33566	−1.568	C_27_H_44_O_3_	Sarsasapogenin
45	5.94	289.07176	289.07239	2.175	C_15_H_14_O_6_	Catechin	130	17.13	837.39142	837.39319	2.110	C_42_H_62_O_17_	Licoricesaponin G2
46	6.17	353.08780	353.08844	1.798	C_16_H_18_O_9_	Chlorogenic acid	131	17.23	271.09648	271.09576	−0.725	C_16_H_14_O_4_	Medicarpin
47	6.41	177.01933	177.01892	−2.327	C_9_H_6_O_4_	Daphnetin	132	17.27	269.08083	269.08029	−2.027	C_16_H_12_O_4_	Formononetin
48	6.76	193.05063	193.05028	−1.824	C_10_H_10_O_4_	Ferulic acid	133	17.42	469.33123	469.33029	−2.016	C_30_H_44_O_4_	Glabrolide isomers
49	6.82	449.10783	449.10599	−4.114	C_21_H_20_O_11_	Cynaroside	134	17.52	187.13396	187.13358	−2.072	C_10_H_20_O_3_	3-Hydroxydecanoic acid
50 *	7.00	417.11800	417.11621	−4.312	C_21_H_20_O_9_	Puerarin	135 *	17.63	821.39650	821.39844	2.351	C_42_H_62_O_16_	Glycyrrhizic Acid
51	7.03	167.03498	167.03445	−3.185	C_8_H_8_O_4_	Vanillic acid	136	17.65	471.34688	471.34561	−2.708	C_30_H_46_O_4_	Enoxolone
52	7.04	593.15119	593.15259	2.355	C_27_H_30_O_15_	Keracyanin Chloride	137	17.80	795.45362	795.45563	2.515	C_42_H_68_O_14_	Eclalbasaponin IV
53	7.04	449.10893	449.11041	3.285	C_21_H_22_O_11_	Eriodictyol-glucoside	138	17.92	471.34688	471.34558	−2.772	C_30_H_46_O_4_	Enoxolone isomers
54	7.04	289.07176	289.07242	2.278	C_15_H_14_O_6_	Epicatechin	139	18.29	431.31558	431.31445	−2.634	C_27_H_42_O_4_	Hecogenin
55	7.22	151.04006	151.03944	−4.154	C_8_H_8_O_3_	2-Hydroxyphenylacetic acid	140	18.59	265.14790	265.14841	1.911	C_12_H_26_O_4_S	Dodecyl sulfate
56	7.27	193.04953	193.04892	−3.187	C_10_H_8_O_4_	Scopoletin	141	18.70	353.13944	353.14014	1.962	C_21_H_22_O_5_	Licochalcone D
57	7.31	337.09289	337.09375	2.549	C_16_H_18_O_8_	Coumaroyl quinic acid	142	18.73	295.09648	295.09564	−2.865	C_18_H_14_O_4_	3-Hydroxymethylenetanshinquinone
58	7.34	433.11292	433.11157	−3.124	C_21_H_20_O_10_	Naringenin-7-glucoside	143	18.74	633.40080	633.40222	2.231	C_36_H_58_O_9_	Eclalbasaponin A
59 *	7.46	301.03537	301.03610	2.405	C_15_H_10_O_7_	Quercetin	144	18.78	805.40159	805.40350	2.366	C_42_H_62_O_15_	Licoricesaponin C2
60	8.05	563.14062	563.14185	2.169	C_26_H_28_O_14_	isoschaftoside	145	18.93	367.11871	367.11938	1.821	C_21_H_20_O_6_	Glycycoumarin
61	8.07	565.15518	565.15344	−3.082	C_26_H_28_O_14_	Schaftoside	146	18.98	807.41724	807.41919	2.41	C_42_H_64_O_15_	Licoricesaponin B2
62	8.10	173.08193	173.08147	2.670	C_8_H_14_O_4_	Suberic acid	147	19.04	305.17583	305.17645	2.203	C_18_H_26_O_4_	Octyl ferulate
63	8.12	313.07176	313.07242	2.104	C_17_H_14_O_6_	Salvianolic acid isomers	148	19.09	311.12778	311.12677	−3.264	C_19_H_18_O_4_	Tanshinone IIB
64	8.12	163.04006	163.03947	−3.664	C_9_H_8_O_3_	P-Coumaric acid	149	19.47	315.08631	315.08505	−4.014	C_17_H_14_O_6_	Pectolinarigenin
65	8.29	319.04484	319.04355	−4.055	C_15_H_10_O_8_	Myricetin	150	19.61	597.30453	597.30609	2.601	C_27_H_51_O_12_P	1-(9Z-octadecenoyl)-sn-glycero-3-phospho-(1′-myo-inositol)
66	8.32	193.05063	193.05031	−1.668	C_10_H_10_O_4_	Isoferulic acid	151	19.64	355.11761	355.11633	−3.618	C_20_H_18_O_6_	Licoflavonol
67	8.32	625.14102	625.14246	2.300	C_27_H_30_O_17_	Quercetin-3-O-neohesperidoside	152	19.64	351.12379	351.12454	2.116	C_21_H_20_O_5_	Gancaonin M
68	8.76	539.11949	539.12085	2.505	C_27_H_24_O_12_	Yunnanec acid D	153	19.68	297.11213	297.11118	−3.216	C_18_H_16_O_4_	Danshenxinkun A
69	8.99	549.16136	549.16235	1.795	C_26_H_30_O_13_	Liguiritigenin-7-O-β-D-apiosyl-4′-O-β-D-glucoside	154	19.88	339.15908	339.15784	−3.673	C_21_H_22_O_4_	Licochalcone A
70	9.02	257.08083	257.07977	−4.144	C_15_H_12_O_4_	Isoliquiritigenin	155	20.11	309.11213	309.11102	−3.609	C_19_H_16_O_4_	Salshenaldehyde
71	9.02	419.13365	419.13202	−3.91	C_21_H_22_O_9_	Liquiritin	156	20.26	335.09249	335.09338	2.336	C_20_H_16_O_5_	Glabrone
72	9.08	479.11840	477.10452	1.406	C_22_H_22_O_12_	Isorhamnetin 3-galactoside	157	20.46	269.04554	269.04610	2.205	_C15_H_10_O_5_	Baicalein
73	9.08	303.05102	303.05154	1.696	C_15_H1_2_O_7_	Taxifolin	158	20.79	279.10157	279.10056	−3.622	C_18_H_14_O_3_	Dihydrotanshinone I
74	9.19	609.14610	609.14697	1.415	C_27_H_30_O_16_	Isorhamnetin-3-O-rutinoside	159	21.01	339.12270	339.12152	−3.48	C_20_H_18_O_5_	Methyltanshinonate
75 *	9.21	611.16066	611.15845	−3.618	C_27_H_30_O_16_	Rutin	160	21.11	393.20603	393.20447	−3.982	C_25_H_28_O_4_	Kanzonol C
76	9.33	521.13006	521.13098	1.758	C_24_H_26_O_13_	Salviaflaside	161	21.76	302.30535	302.30405	−4.320	C_18_H_39_NO_2_	2,2′-(Tetradecylimino)diethanol
77 *	9.47	317.06557	317.06439	−3.751	C_16_H_12_O_7_	Isorhamnetin	162	21.83	468.30846	468.30676	−3.642	C_22_H_46_NO_7_P	1-Myristoyl-sn-glycero-3-phosphocholine
78	9.55	465.10275	465.10120	−3.338	C_21_H_20_O_12_	Isoquercitin	163	21.89	297.14852	297.14725	−4.277	C_19_H_20_O_3_	Cryptotanshinone
79	9.63	447.09328	447.09421	2.07	C_21_H_20_O_11_	5-O-β-D-glucosyl-4′,7-dihydroxycoumarin	164	22.18	277.08592	277.08493	−3.576	C_18_H_12_O_3_	Tanshinone I
80	9.80	517.13405	517.13251	−2.983	C_25_H_24_O_12_	Isochlorogenic acid B	165	22.27	452.27826	452.27936	2.427	C_21_H_44_NO_7_P	1-Palmitoyl-2-hydrOxy-sn-glycero-3-phosphoethanolamine
81	9.94	539.11840	539.11707	−1.332	C_27_H_22_O_12_	Lithospermic acid	166	22.56	293.11722	293.11618	−3.551	C_19_H_16_O_3_	Dehydrotanshinone II A
82	10.00	315.05102	315.05151	0.484	C_16_H_12_O_7_	Eupafolin	167	22.76	281.15360	281.15265	−3.402	C_19_H_20_O_2_	Dehydromiltirone
83	10.01	551.10314	551.10150	−3.314	C_24_H_22_O_15_	Quercetin 3-O-malonylglucoside	168	23.13	295.13280	295.13156	−1.31	C_19_H_18_O_3_	Tanshinone IIA
84 *	10.16	517.13405	517.13245	−3.099	C_25_H_24_O_12_	isochlorogenic acid A	169	23.34	283.16925	283.16824	−3.59	C_19_H_22_O_2_	Miltrione
85	10.22	492.31670	492.31534	−2.779	C_24_H_45_NO_9_	Morusimic acid C isomers I	170	24.42	455.35306	455.35406	2.177	C_21_H_20_O_6_	Oleanolic acid

* Compared with standard compounds.

## Data Availability

The original contributions presented in this study are included in the article/Supplementary Materials. Further inquiries can be directed to the corresponding authors.

## Supplementary Materials

The following supporting information can be downloaded at https://www.mdpi.com/article/10.3390/ph19030459/s1, Table S1: Identification of chemical components in XBKF using UHPLC-Q-Exactive Orbitrap MS; Table S2: Detailed information of the 22 reference standards. Figure S1: High-resolution extraction ion chromatograms (TIC) of XBKF capsules in positive-ion (P1,P2) and negative-ion (N1,N2) modes.
