# Supplementary material for: Rapid Analysis of the Chemical Composition of Xiaoban Kangfu Capsules Based on UHPLC-Q-Exactive Orbitrap MS/MS Combined with Molecular Networks
Source: Pharmaceuticals (Basel). 2026 Mar 11;19(3):459. doi: 10.3390/ph19030459 (PMC13028765; doi:10.3390/ph19030459)
Supplement: Supplementary file 1 [file pharmaceuticals-19-00459-s001.zip › pharmaceuticals-4128503-supplementary.pdf]

Table S1 Identification of chemical components in XBKF by UHPLC-Q-Exactive Orbitrap MS.

Peak	t_R(min)	Theoretical Mass *m/z*	Experimental Mass *m/z*	Error (ppm)	MS/MS fragment	Formula	MS/MS fragment（*m/z*）	Identification	Types
1	0.69	259.02244	259.02286	1.615	$[M-H]^-$	$C_6H_{13}O_9P$	MS^2[259]:96.9590(100),96.9685(73),78.9578(44),138.9792(3)	Glucose-6-Phosphate	Carbohydrates
2	0.75	665.21458	665.21600	2.133	$[M-H]^-$	$C_{24}H_{42}O_{21}$	MS^2[665]:89.0233(100),101.0234(63),179.0557(49),59.0126(41),71.0126(37)	Stachyose	Carbohydrates
3	0.78	387.11441	387.11526	2.031	$[M-H]^-$	$C_{13}H_{24}O_{13}$	MS^2[387]:89.0232(100),341.1094(48),119.0341(32),179.0556(14),161.0448(8）	2,3,4,5,6-pentahydroxy-7-[(2S,3R,4S,5S,6R)-3,4,5-trihydroxy-6-(hydroxymethyl)oxan-2-yl]oxyheptanoic acid	Other categories
4*	0.79	341.10893	341.10950	1.657	$[M-H]^-$	$C_{12}H_{22}O_{11}$	MS^2[341]:89.0232(100),59.0126(63),71.0126(45),101.0233(34),119.0340(31)	Sucrose	Carbohydrates
5*	0.80	179.05611	179.05568	-2.409	$[M-H]^-$	$C_6H_{12}O_6$	MS^2[179]:59.0126(100),71.0126(74),89.0233(57),101.0235(16)	D-Galactose	Carbohydrates
6	0.81	191.05611	191.05576	-1.839	$[M-H]^-$	$C_7H_{12}O_6$	MS^2[191]:111.0077(100),87.0076(39),85.0283(27)	Quinic acid	Organic acid
7*	0.82	149.04554	149.04485	-6.074	$[M-H]^-$	$C_5H_{10}O_5$	MS^2[149]:89.0232(100),59.0126(80),75.0075(28)	D-ribose	Carbohydrates
8	0.82	195.05102	195.05072	-1.722	$[M-H]^-$	$C_6H_{12}O_7$	MS^2[195]:195.0507(100),129.0	Gluconic acid	Organic acid

							185(66),87.0076(26),99.0077(22),59.0126(18)		
9	0.85	177.04046	177.04001	-2.549	$[M-H]^-$	$C_6H_{10}O_6$	MS^2[177]:177.0399(67),129.0184(62),99.0076(53),59.0126(45),89.0232(42)	Gluconolactone	Organic acid
10	0.86	209.03029	209.03012	-0.816	$[M-H]^-$	$C_6H_{10}O_8$	MS^2[209]:85.0283(100),129.0184(56),209.0665(49),71.0126(13)	Mucic acid	Other categories
11	0.87	266.12342	266.12234	-4.07	$[M+H]^+$	$C_{10}H_{19}NO_7$	MS^2[266]:248.1117(100),230.1013(50),194.0806(15)	Fructose-Ethylglycine	Other categories
12	0.87	161.04554	161.04495	-3.705	$[M-H]^-$	$C_6H_{10}O_5$	MS^2[161]:101.0234(100),59.0126(52),99.0441(15),57.0332(9),161.0448(7)	3-Hydroxy-3-methylglutaric acid	Organic acid
13*	0.88	133.01424	133.01343	-6.289	$[M-H]^-$	$C_4H_6O_5$	MS^2[133]:115.0027(100),133.0134(40),71.0126(37)	Malic acid	Organic acid
14	0.88	278.12342	278.12238	-3.77	$[M+H]^+$	$C_{11}H_{19}NO_7$	MS^2[278]:242.1012(100),260.1117(85),232.1170(46),214.1065(25)	Fructose-Proline	Other categories
15	0.88	138.05495	138.05449	-3.369	$[M+H]^+$	$C_7H_7NO_2$	MS^2[138]:138.0544(100),94.0652(3),110.0598(2)	2-Aminobenzoic acid	Organic acid
16	0.88	290.08813	290.08862	1.656	$[M-H]^-$	$C_{11}H_{17}NO_8$	MS^2[290]:128.0344(100),200.0561(19),170.0453(8)	N-Fructosyl pyroglutamate	Other categories
17	0.91	173.00916	173.00864	-3.012	$[M-H]^-$	$C_6H_6O_6$	MS^2[173]:111.0077(100),85.0283(19)	Cis -Aconitic acid	Other categories
18*	0.91	191.01972	191.01933	-2.072	$[M-H]^-$	$C_6H_8O_7$	MS^2[191]:111.0077(100),87.0076(39),85.0283(28),191.0193(8	Citric acid	Other categories

							)		
19	0.93	130.08625	130.08589	-2.807	$[M+H]^+$	$C_6H_{11}NO_2$	MS^2[130]:84.0810(100),130.0858(45),112.0753(1)	Pipecolic acid	Organic acid
20	1.18	124.0393	124.03903	-2.217	$[M+H]^+$	$C_6H_5NO_2$	MS^2[124]:96.0444(100),80.0497(54),78.0340(5)	Nicotinic acid	Organic acid
21	1.18	290.08813	290.08871	1.966	$[M-H]^-$	$C_{11}H_{17}NO_8$	MS^2[290]:128.0344(100),200.0562(20)	Fructose-Pyrrolidonecarboxylic acid	Other categories
22	1.23	344.13399	344.13266	-3.374	$[M+H]^+$	$C_{15}H_{21}NO_8$	MS^2[344]:280.1167(98),308.1115(59),326.1219(18)	Fructose-tyrosine	Other categories
23	1.23	182.08116	182.08061	-3.074	$[M+H]^+$	$C_9H_{11}NO_3$	MS^2[182]:136.0751(100),123.0437(41),96.0444(27),119.0488(22)	L-Tyrosine	Other categories
24	1.25	147.02989	147.02925	-4.398	$[M-H]^-$	$C_5H_8O_5$	MS^2[147]:147.0291(100),85.0283(80),129.0184(58),103.0390(22),101.0233(10)	α-Hydroxyglutaric acidα	Organic acid
25	1.29	117.01933	117.01845	-7.537	$[M-H]^-$	$C_4H_6O_4$	MS^2[117]:73.0282(100),117.0183(27),99.0076(11)	Succinic Acid	Organic acid
26*	1.33	152.05668	152.05615	-3.528	$[M+H]^+$	$C_5H_5N_5O$	MS^2[152]:252.0561(100),110.0347(16),135.0296(10)	Guanine	Other categories
27	1.38	292.14017	292.14087	2.378	$[M-H]^-$	$C_{12}H_{23}NO_7$	MS^2[292]:130.0864(100),101.0233(9),110.0964(3),172.0974(3)	Fructose-L-isoleucine	Other categories
28	1.73	169.01424	169.01373	-3.056	$[M-H]^-$	$C_7H_6O_5$	MS^2[169]:125.0235(100),126.0266(2)	Gallic acid	Phenolic acids
29	2.19	328.13907	328.13770	-4.201	$[M+H]^+$	$C_{15}H_{21}NO_7$	MS^2[328]:264.1218(100),292.1	Fructose-phenyla	Other

							164(52),246.1113(26)310.1268(16)	lanine	categories
30	2.19	329.08780	329.08859	2.384	[M-H]$^-$	$C_{14}H_{18}O_9$	MS^2[329]:109.0285(100),167.0343(89),165.0187(18),59.0125(11),123.0442(10),123.0442(10),71.0126(8),101.0232(8)	Phenylacetic acid + 2O, O-Hex	Other categories
31	2.27	166.08625	166.08572	-3.222	[M+H]$^+$	$C_9H_{11}NO_2$	MS^2[166]:120.0805(100),131.0486(4),103.0541(3),149.0589(1)	L-Phenylalanine	Other categories
32	2.53	220.11794	220.11717	-3.540	[M+H]$^+$	$C_9H_{17}NO_5$	MS^2[220]:90.0551(100),202.1065(23),184.0961(18),116.0340(14)	Pantothenic acid	Other categories
33	2.54	218.10339	218.10336	-0.165	[M-H]$^-$	$C_9H_{17}NO_5$	MS^2[218]:88.0392(100),146.0815(56)	D-pantothenic acid	Other categories
34	2.56	197.04554	197.04530	-1.252	[M-H]$^-$	C9H10O5	MS^2[197]:135.0443(100),179.0344(71),123.0442(67)	Salvianic acid A.	Phenolic acids
35	2.58	417.08271	417.08142	-3.117	[M-H]$^-$	$C_{20}H_{18}O_{10}$	MS^2[417]:219.0275(100),197.0453(21),179.0346(11)	Salvianolic acid D	Phenolic acids
36	2.59	153.01933	153.01868	-0.652	[M-H]$^-$	$C_7H_6O_4$	MS^2[153]:109.0285(100),153.0186(97),108.0207(2),81.0334(1)	Gentisic acid	Phenolic acids
37	3.31	153.01933	153.01868	-4.261	[M-H]$^-$	$C_7H_6O_4$	MS^2[153]:109.0285(100),153.0187(32),113.9844(1),123.0442(1)	Protocatechuic acid	Phenolic acids
38*	4.31	181.04953	181.04884	-3.84	[M+H]$^+$	$C_9H_8O_4$	MS^2[181]:163.0383(100),145.0278(21),135.0435(21)	Caffeic acid	Phenolic acids

39*	4.32	353.08780	353.08853	2.052	[M-H]⁻	$C_{16}H_{18}O_9$	MS^2[353]:191.0557(100),179.0345(71),135.0443(36),173.0452(3),161.0239(3)	Cryptochlorogenic acid	Phenolic acids
40*	4.91	137.02441	137.02371	-5.162	[M-H]⁻	$C_7H_6O_3$	MS^2[137]:137.0244(100),93.0339(9)	4-hydroxybenzoic acid	Phenolic acids
41	5.07	175.06119	175.06073	-2.666	[M-H]⁻	$C_7H_{12}O_5$	MS^2[175]:115.0391(100),175.0607(55),113.0598(37),85.0647(36),146.9602(1)	2-Isopropylmalic acid	Organic acid
42	5.07	165.05571	165.05516	-3.377	[M-H]⁻	$C_9H_{10}O_3$	MS^2[165]:121.0650(100),93.0334(87),119.0493(22)	2-phenoxypropanoicacid	Other categories
43	5.54	577.13514	577.13696	3.137	[M-H]⁻	$C_{15}H_{11}O_6$	MS^2[577]:125.0235(100),289.07239(52),161.0238(35),407.0778(24)	Cyanidin isomer	Flavanol
44*	5.54	577.13514	577.13696	1.811	[M-H]⁻	$C_{30}H_{26}O_{12}$	MS^2[577]:125.0235(100),289.0723(52),245.0820(38),161.0238(35),407.0778(24),137.0235(20),109.0285(11)	Procyanidin B1	Flavanol
45	5.94	289.07176	289.07239	2.175	[M-H]⁻	$C_{15}H_{14}O_6$	MS^2[289]:109.0285(100),245.0822(83),125.0235(75),203.0711(73),179.0345(35)	Catechin	Flavanol
46	6.17	353.08780	353.08844	1.798	[M-H]⁻	$C_{16}H_{18}O_9$	MS^2[353]:191.0558(100),173.0451(58),179.0346(44),135.0443(13)	Chlorogenic acid	Phenolic acids
47	6.41	177.01933	177.01892	-2.327	[M-H]⁻	$C_9H_6O_4$	MS^2[177]:133.0286(100),105.0335(31),149.0237(9)	Daphnetin	Coumarins
48	6.76	193.05063	193.05028	-1.824	[M-H]⁻	$C_{10}H_{10}O_4$	MS^2[193]:134.0365(100),178.0	Ferulic acid	Phenolic acids

							266(63),193.0504(13),149.0601(9		
49	6.82	449.10783	449.10599	-4.114	$[M+H]^+$	$C_{21}H_{20}O_{11}$	MS^2[449]:287.0537(100),285.0409(55),288.0570(8)	Cynaroside	Flavonoids
50*	7.00	417.11800	417.11621	-4.312	$[M+H]^+$	$C_{21}H_{20}O_9$	MS^2[417]:297.0744(100),267.0639(58),321.0742(29),307.0949(21),363.0845(17),335.0901(11)	Puerarin	Isoflavonoids
51	7.03	167.03498	167.03445	-3.185	$[M-H]^-$	$C_8H_8O_4$	MS^2[167]:167.0343(100),123.0442(53),108.0206(24),152.0107(16)	Vanillic acid	Phenolic acids
52	7.04	593.15119	593.15259	2.355	$[M-H]^-$	$C_{27}H_{30}O_{15}$	MS^2[593]:284.0330(100),285.0408(55)	Keracyanin Chloride	Flavonoids
53	7.04	449.10893	449.11041	3.285	$[M-H]^-$	$C_{21}H_{22}O_{11}$	MS^2[449]:269.0461(45),259.0619(23),59.0125(19),125.0234(15),287.0565(8)	Eriodictyol-glucoside	flavanone
54	7.04	289.07176	289.07242	2.278	$[M-H]^-$	$C_{15}H_{14}O_6$	MS^2[289]:109.0285(100),125.0235(82),245.0821(83),203.0711(83),205.0503(54),187.0396(19)	Epicatechin	Flavanol
55	7.22	151.04006	151.03944	-4.154	$[M-H]^-$	$C_8H_8O_3$	MS^2[151]:107.0491(100),151.0393(63)	2-Hydroxyphenylacetic acid	Phenolic acids
56	7.27	193.04953	193.04892	-3.187	$[M+H]^+$	$C_{10}H_8O_4$	MS^2[193]:193.0489(100),134.0	Scopoletin	Coumarins
57	7.31	337.09289	337.09375	2.549	$[M-H]^-$	$C_{16}H_{18}O_8$	MS^2[337]:191.0557(100),93.0334(23),173.0450(15),163.0394(14)	Coumaroyl quinic acid	Phenolic acids

58	7.34	433.11292	433.11157	-3.124	$[M+H]^+$	$C_{21}H_{20}O_{10}$	MS^2[433]:271.0589(100),272.06229(8)	Naringenin-7-glucoside	flavanone
59*	7.46	301.03537	301.03610	2.405	$[M-H]^-$	$C_{15}H_{10}O_7$	MS^2[301]:301.0358(100),257.0459(75),151.0029(41),149.0237(2),273.0408(2)	Quercetin	Flavonols
60	8.05	563.14062	563.14185	2.169	$[M-H]^-$	$C_{26}H_{28}O_{14}$	MS^2[563]:353.0672(100),383.0779(70),443.0992(17)，413.0882(10),365.0668(10)	isoschaftoside	Flavonoids
61	8.07	565.15518	565.15344	-3.082	$[M+H]^+$	$C_{26}H_{28}O_{14}$	MS^2[565]:391.0800(100)325.0695(93),379.0791(89),349.0688(84),295.0586(70),351.0835(20)	Schaftoside	Flavonoids
62	8.10	173.08193	173.08147	2.670	$[M-H]^-$	$C_8H_{14}O_4$	MS^2[173]:111.0805(100),173.0814(41),129.0912(7)	Suberic acid	Organic acid
63	8.12	313.07176	313.07242	2.104	$[M-H]^-$	$C_{17}H_{14}O_6$	MS^2[313]:109.0285(100),159.0446(16),147.0444(11)	Salvianolic acid isomers	Phenolic acids
64	8.12	163.04006	163.03947	-3.664	$[M-H]^-$	$C_9H_8O_3$	MS^2[163]:119.0492(100),163.0394(14)	P - Coumaric acid	Coumarins
65	8.29	319.04484	319.04355	-4.055	$[M+H]^+$	$C_{15}H_{10}O_8$	MS^2[319]:319.0433(100),273.0381(3),245.0432(2),301.1531(2)	Myricetin	Flavonols
66	8.32	193.05063	193.05031	-1.668	$[M-H]^-$	$C_{10}H_{10}O_4$	MS^2[193]:149.0238(100),134.0365(52),178.0269(50)	Isoferulic acid	Phenolic acids
67	8.32	625.14102	625.14246	2.300	$[M-H]^-$	$C_{27}H_{30}O_{17}$	MS^2[6225]:300.028(100),302.0388(47),151.0029(7),271.0248(4)	Quercetin-3-O-neohesperidoside	Flavonols

68	8.76	539.11949	539.12085	2.505	$[M-H]^{-}$	$C_{27}H_{24}O_{12}$	MS^2[539]:161.0238(100),135.0443(97),197.0452(65),179.0345(52),297.0777(28)	Yunnanec acid D	Triterpenoids
69	8.99	549.16136	549.16235	1.795	$[M-H]^{-}$	$C_{26}H_{30}O_{13}$	MS^2[549]:255.0666(100),135.0079(25),297.0771(2)549.1628(1)	Liguiritigenin-7-O-β-D-apiosyl-4'-O-β-D-glucoside	flavanone
70	9.02	257.08083	257.07977	-4.144	$[M+H]^{+}$	$C_{15}H_{12}O_4$	MS2[257]:137.0228(100),147.0434(47),257.0796(41),211.0745(12),130.1422(1),163.0383(5)	Isoliquiritigenin	Chalcones
71	9.02	419.13365	419.13202	-3.91	$[M+H]^{+}$	$C_{21}H_{22}O_9$	MS^2[419]:257.0797(100),137.0228(36),147.0434(22),85.0286(17),239.0694(6),163.0383(3),109.0282(2)	Liquiritin	flavanone
72	9.08	479.11840	477.10452	1.406	$[M-H]^{-}$	$C_{22}H_{22}O_{12}$	MS^2[479]:314.0439(100),477.1058(2)	Isorhamnetin 3-galactoside	Flavonols
73	9.08	303.05102	303.05154	1.696	$[M-H]^{-}$	$C_{15}H1_2O_7$	MS^2[303]:125.0235(100),285.0408(22)	Taxifolin	flavanone
74	9.19	609.14610	609.14697	1.415	$[M-H]^{-}$	$C_{27}H_{30}O_{16}$	MS^2[609]:300.0280(100),301.0358(36),271.0255(6),151.0029(6)	Isorhamnetin-3-O-rutinoside	Flavonols
75*	9.21	611.16066	611.15845	-3.618	$[M+H]^{+}$	$C_{27}H_{30}O_{16}$	MS^2[611]:303.0485(100),85.0286(24),71.0495(15),287.0537(4),129.0542(3)	Rutin	Flavonols
76	9.33	521.13006	521.13098	1.758	$[M-H]^{-}$	$C_{24}H_{26}O_{13}$	MS^2[521]:161.0237(100),197.0	Salviaflaside	Phenolic acids

							452(28),179.0345(26),323.0778(19),135.0443(12)		
77*	9.47	317.06557	317.06439	-3.751	$[M+H]^+$	$C_{16}H_{12}O_7$	MS^2[317]:317.0643(100),302.0408(15),285.0382(6),274.0455(2),153.0162(4)	Isorhamnetin	Flavonols
78	9.55	465.10275	465.10120	-3.338	$[M+H]^+$	$C_{21}H_{20}O_{12}$	MS^2[465]:303.0486(100),304.0520(9),85.0286(13),97.0284(4),	Isoquercitin	Flavonols
79	9.63	447.09328	447.09421	2.07	$[M-H]^-$	$C_{21}H_{20}O_{11}$	MS^2[447]:285.0410(100),284.0331(31),286.0441(3)	5-O-β-D-glucosyl-4',7-dihydroxycoumarin	Coumarins
80	9.80	517.13405	517.13251	-2.983	$[M+H]^+$	$C_{25}H_{24}O_{12}$	MS^2[517]:163.0383(100),145.0278(3),117.0332(1)，181.0487(1)	Isochlorogenic acid B	Phenolic acids
81	9.94	539.11840	539.11707	-1.332	$[M+H]^+$	$C_{27}H_{22}O_{12}$	MS^2[539]:139.0386(100),251.0696(52),135.0437(38),163.0385(21),295.0592(17),181.0490(16),231.0283(16),187.0385(10)	Lithospermic acid	Phenolic acids
82	10.00	315.05102	315.05151	0.484	$[M-H]^-$	$C_{16}H_{12}O_7$	MS^2[315]:300.0280(100),315.0516(79),301.0313(3)	Eupafolin	Flavonoids
83	10.01	551.10314	551.10150	-3.314	$[M+H]^+$	$C_{24}H_{22}O_{15}$	MS^2[551]:303.0487(100),85.0286(18),127.0386(15),109.0283(12),81.0338(9)	Quercetin 3-O-malonylglucoside	Flavonols
84*	10.16	517.13405	517.13245	-3.099	$[M+H]^+$	$C_{25}H_{24}O_{12}$	MS^2[517]:163.0383(100),145.0279(1),117.0333(1)	isochlorogenic acid A	Phenolic acids
85	10.22	492.31670	492.31534	-2.779	$[M+H]^+$	$C_{24}H_{45}NO_9$	MS^2[492]:330.2626(100),312.2	Morusimic acid	Other

							521(39),294.2422(5),474.3036(2),	C isomers I	categories
86*	10.37	285.04046	285.04092	1.609	[M-H]$^{-}$	$C_{15}H_{10}O_6$	MS^2[285]:285.0409(100),241.0506(3),151.0030(2),133.0288(2)	Luteolin	Flavonoids
87	10.47	433.11402	433.11472	1.616	[M-H]$^{-}$	$C_{21}H_{22}O_{10}$	MS^2[433]:271.0617(100),151.0030(29),119.0493(5)	Naringenin -7-O-glucoside	flavanone
88	10.52	209.08083	209.08026	-2.752	[M+H]$^{+}$	$C_{11}H_{12}O_4$	MS^2[209]:55.0184(100),191.0696(38),145.0279(33),209.0802(16),163.0747(14),135.0436,181.0853(11),117.0333(9),107.0490(8)	Ethyl caffeate	Phenolic acids
89	10.60	301.07066	301.06964	-3.403	[M+H]$^{+}$	$C_{16}H_{12}O_6$	MS^2[301]:301.06952(100),286.04620(6)	Fallacinol	Quinones
90	10.65	1079.52687	1079.52441	-2.288	[M+H]$^{+}$	$C_{51}H_{82}O_{24}$	MS^2[1079]:85.0286(100),97.0284(44),127.0386(36),299.2356(21),395.2930(15)	Terrestrosin K	Other categories
91	10.67	654.36953	654.36780	-2.646	[M+H]$^{+}$	$C_{30}H_{55}NO_{14}$	MS^2[654]:330.2624(100),312.2523(40),268.2625(35),250.2516(23),492.3126(13)	Morusimic acid C isomer+Glu	Other categories
92	10.71	161.02441	161.02371	-4.393	[M-H]$^{-}$	$C_9H_6O_3$	MS^2[161]:161.0237(100),133.0286(79)	7-Hydroxycoumarin	Coumarins
93*	10.71	359.07724	359.07797	2.031	[M-H]$^{-}$	$C_{18}H_{16}O_8$	MS^2[359]:161.0238(100),197.0452(390,179.0345(26),72.9919(16),135.0443(8)	Rosmarinic Acid	Phenolic acids
94	10.87	517.13405	517.13257	-2.867	[M+H]$^{+}$	$C_{25}H_{24}O_{12}$	MS^2[517]:163.0384(100)145.0	Isochlorogenic	Phenolic acids

							279(9),89.0599(6),319.0800(1)	acid C	
95	10.98	537.10384	537.10492	1.994	$[M-H]^-$	$C_{27}H_{22}O_{12}$	MS^2[537]:295.0616(100),109.0284(77),339.0513(16),321.0408(19)	Salvianolic acid H/J	Phenolic acids
96	10.98	493.11402	493.11508	2.15	$[M-H]^-$	$C_{26}H_{22}O_{10}$	MS^2[493]:109.0285(100),185.0240(88),295.0616(39),197.0452(12)	Salvianolic acid A	Phenolic acids
97	11.02	341.06557	341.06454	-3.047	$[M+H]^+$	$C_{18}H_{12}O_7$	MS^2[341]:249.0536(100),277.0484(62),295.0588(48),323.0538(8)	Salvianolic acid G	Phenolic acids
98*	11.38	329.10306	329.10361	1.667	$[M-H]^-$	$C_{18}H_{18}O_6$	MS^2[329]:59.01260(100),269.08203(13)	Acetylshikonin	Quinones
99	11.68	551.17591	551.17450	2.531	$[M+H]^+$	$C_{26}H_{30}O_{13}$	MS^2[551]:257.0800(100),137.0229(29),147.0436(19),85.0287(3)	Liquiritin apioside	flavanone
100	11.75	139.03897	137.02286	-3.361	$[M+H]^+$	$C_7H_6O_3$	MS^2[137]:111.0441(100),93.0337(14),139.0386(19),65.0391(10),134.0598(1),121.0394(2),83.0495(2)	Protocatechualdehyde	Phenolic acids
101	11.76	717.14610	717.14752	1.885	$[M-H]^-$	$C_{36}H_{30}O_{16}$	MS^2[717]:321.0410(100),295.0615(25),185.0240(19),109.0285(15)	Salvianolic acid B	Phenolic acids
102	12.10	419.13365	419.13248	2.812	$[M+H]^+$	$C_{21}H_{22}O_9$	MS^2[419]:257.0802(100),137.0230(31),147.0437(21),239.0696(6),211.0748(5)	Isoliquiritin	Chalcones
103	12.48	551.11949	551.12085	3.466	$[M-H]^-$	$C_{28}H_{24}O_{12}$	MS^2[551]:294.0539(100),197.0	Salvianolic acid	Phenolic acids

							453(99),179.0345(89),72.9919(81),135.0443(61)	isomers	
104*	12.64	255.06628	255.06676	1.874	$[M-H]^-$	$C_{15}H_{12}O_4$	MS^2[255]:119.0493(100),135.0079(71),91.0178(10)	Liquiritigenin	flavanone
105	12.64	285.07684	285.07751	2.326	$[M-H]^-$	$C_{16}H_{14}O_5$	MS^2[285]:150.03156(100),270.0539(50)	Licochalcone B	Chalcones
106	12.74	253.05063	253.05112	1.928	$[M-H]^-$	$C_{15}H_{10}O_4$	MS^2[253]:117.0336(100),153.0187(40),135.0080(41),91.0178(8)	Daidzein	Isoflavonoids
107	12.80	201.11323	201.11310	-0.685	$[M-H]^-$	$C_{10}H_{18}O_4$	MS^2[201]:139.1120(100),201.1129(72),183.1022(37)	3-tert-Butyladipic acid	Organic acid
108	12.87	285.04046	285.04120	2.592	$[M-H]^-$	$C_{15}H_{10}O_6$	MS^2[285]:285.04095(100),257.04575(5),241.05070(1)	Citreorosein	Quinones
109	12.90	373.09289	373.09366	2.062	$[M-H]^-$	$C_{19}H_{18}O_8$	MS^2[373]:135.0443(100),179.0343(3)	Methyl rosmarinate	Phenolic acids
110	13.05	299.05611	299.05676	2.169	$[M-H]^-$	$C_{16}H_{12}O_6$	MS^2[299]:299.0566(100),284.0331(77),285.0358(4)	Hispidulin	Flavonoids
111	13.11	551.11949	551.12122	3.112	$[M-H]^-$	$C_{28}H_{24}O_{12}$	MS^2[551]:321.0411(100),353.0670(18),339.0517(4)	Paederosidic acid methyl ester	Phenolic acids
112	13.15	556.21772	556.21808	0.643	$[M+H]^+$	$C_{29}H_{33}NO_{10}$	MS^2[556]:269.0806(100),270.0841(9),288.1444(2)	Isoflavone base + 1O, 1MeO, O-Hex+C7H12NO	Isoflavonoids
113	13.37	339.10744	339.10745	0.018	$[M+H]^+$	$C_{16}H_{18}O_8$	MS^2[339]:177.0546(100),145.0284(33),137.0594	Gerberinside	Coumarins
114	13.48	431.13365	431.13159	-4.789	$[M+H]^+$	$C_{22}H_{22}O_9$	MS^2[431]:269.0801(100),254.0	Ononin	Isoflavonoids

							568(1)		
115*	13.58	285.07575	285.07587	0.421	$[M+H]^+$	$C_{16}H_{12}O_5$	MS^2[285]:285.07581(100)，286.07916(10),253.0497(8),225.0548(6),286.0459(2)	Physcion	Quinones
116	13.58	285.07575	285.07580	0.421	$[M+H]^+$	$C_{16}H_{12}O_5$	MS^2[285]:285.0760(100),270.0525(85),253.0497(6)	Wogonin	Flavonoids
117	13.61	441.20201	441.20233	0.718	$[M+H]^+$	$C_{24}H_{28}N_2O_6$	MS2[441]:177.0548(100),145.0285(25),265.1546(6)	Diferuloyl putrescine	Phenolic acids
118*	13.91	271.06009	271.06042	1.181	$[M+H]^+$	$C_{15}H_{10}O_5$	MS^2[271]:271.06049(100),229.05003(16),201.05498(5)，243.0652(1)	Emodin	Quinones
119	13.92	307.07243	307.07303	1.954	$[M-H]^-$	$C_{17}H_{12}N_2O_4$	MS^2[307]:233.0721(100),263.0830(45),205.0768(15),191.0612(2)	Flazin	Other categories
120	14.29	315.04992	315.05057	2.034	$[M+H]^+$	$C_{16}H_{10}O_7$	MS^2[315]:287.0557(100),300.0636(59),297.0401(46),269.0452(23)	Wedelolactone	Coumarins
121	14.45	461.10893	461.11002	2.354	$[M-H]^-$	$C_{22}H_{22}O_{11}$	MS^2[461]:299.0565(100),284.0331(17),300.0603(3)	Peonidin-3-O-beta-galactoside	Flavonoids
122	14.93	271.06119	271.06189	2.558	$[M-H]^-$	$C_{15}H_{12}O_5$	MS^2[271]:151.0030(100),119.0493(48),271.0618(35),107.0128(17),93.0335(13),177.0190(13)	Naringenin	flavanone
123	15.04	433.33123	433.33319	4.508	$[M+H]^+$	$C_{27}H_{44}O_4$	MS^2[433]:289.2174(100),161.1332(82),253.1961(33),69.0708(26),271.2070(22)	Gitogenin	Other categories

124	15.61	271.09648	271.09741	3.410	$[M+H]^+$	$C_{16}H_{14}O_4$	MS^2[271]:121.0290(100),229.0867(25),107.0499(19),177.0553(18),123.0446(14)	Retrochalcone	Chalcones
125	15.66	875.41044	875.41248	2.325	$[M-H]^-$	$C_{42}H_{68}O_{17}S$	MS^2[875]:241.0025(100),875.4095(28),713.3546(21)	Eclalbasaponin VI	Triterpenoids
126	15.93	445.11402	445.11496	2.112	$[M-H]^-$	$C_{22}H_{22}O_{10}$	MS^2[445]:283.0616(100),240.0428(16),284.0651(4),268.0381(3)	Calycosin-7-O-β-D-glucoside	Isoflavonoids
127	15.97	313.07176	313.07248	2.295	$[M-H]^-$	$C_{17}H_{14}O_6$	MS^2[313]:161.023(100),151.0393(9),133.0286(6)	Salvianolic acid F	Phenolic acids
128	16.16	469.33123	469.33209	1.819	$[M+H]^+$	$C_{30}H_{44}O_4$	MS^2[469]:175.1483(100),187.1483(83),149.1326(74),189.1639(66),135.1169(63),173.1325(59),217.1590(44),121.1014(44)	Glabrolide	Triterpenoids
129	16.82	417.33632	417.33566	-1.568	$[M+H]^+$	$C_{27}H_{44}O_3$	MS^2[417]:273.2207(100),161.1322(87),255.210(66),167.0700(53),69.0704(20)	Sarsasapogenin	Triterpenoids
130	17.13	837.39142	837.39319	2.110	$[M-H]^-$	$C_{42}H_{62}O_{17}$	MS^2[837]:113.0234(100),193.0350(45),351.0575(45),175.0244(19)	Licoricesaponin G2	Triterpenoids
131	17.23	271.09648	271.09576	-0.725	$[M+H]^+$	$C_{16}H_{14}O_4$	MS^2[271]:137.0593(100),123.0438(10),161.0593(8),147.0437(4)	Medicarpin	Isoflavonoids
132	17.27	269.08083	269.08029	-2.027	$[M+H]^+$	$C_{16}H_{12}O_4$	MS^2[269]:269.0803(100),254.0569(6),213.0906(4),237.0541(3)	Formononetin	Isoflavonoids

133	17.42	469.33123	469.33029	-2.016	$[M+H]^+$	$C_{30}H_{44}O_4$	MS^2[469]:189.1634(100),95.0857(74),175.1477(68),235.1687(65)	Glabrolide isomers	Triterpenoids
134	17.52	187.13396	187.13358	-2.072	$[M-H]^-$	$C_{10}H_{20}O_3$	MS^2[187]:141.8674(100),125.09641(10)，59.0126(1)	3-Hydroxydecanoic acid	Other categories
135*	17.63	821.39650	821.39844	2.351	$[M-H]^-$	$C_{42}H_{62}O_{16}$	MS^2[821]:113.0234(100),193.0350(47),85.0283(41),351.0579(38),175.0243(24)	Glycyrrhizic Acid	Triterpenoids
136	17.65	471.34688	471.34561	-2.708	$[M+H]^+$	$C_{30}H_{46}O_4$	MS^2[471]:471.3457(100),317.2104(45),235.1687(46),189.1633(70),95.0857(45),453.3346(17),425.3401(16),263.1634(15)	Enoxolone	Triterpenoids
137	17.80	795.45362	795.45563	2.515	$[M-H]^-$	$C_{42}H_{68}O_{14}$	MS^2[795]:633.4024(100),471.3489(47),161.0450(24),407.3323(3)	Eclalbasaponin IV	Triterpenoids
138	17.92	471.34688	471.34558	-2.772	$[M+H]^+$	$C_{30}H_{46}O_4$	MS^2[471]:189.1633(100),95.0857(65),175.1476(63),121.1011(41),107.0855(28)	Enoxolone isomers	Triterpenoids
139	18.29	431.31558	431.31445	-2.634	$[M+H]^+$	$C_{27}H_{42}O_4$	MS^2[431]:69.0704(100),299.2361(43),187.1477(35),133.1008(30),281.2255(25)	Hecogenin	Other categories
140	18.59	265.14790	265.14841	1.911	$[M-H]^-$	$C_{12}H_{26}O_4S$	MS^2[265]:265.1484(100),266.1519(4)	Dodecyl sulfate	Organic acid
141	18.70	353.13944	353.14014	1.962	$[M-H]^-$	$C_{21}H_{22}O_5$	MS^2[353]:150.0315(86),338.1168(48)，353.1404(13)	Licochalcone D	Chalcones
142	18.73	295.09648	295.09564	-2.865	$[M+H]^+$	$C_{18}H_{14}O_4$	MS^2[295]:277.0850(100),249.0	3-Hydroxymethy	Triterpenoids

							901(98),267.1005(10)	lenetanshinquinone	
143	18.74	633.40080	633.40222	2.231	$[M-H]^-$	$C_{36}H_{58}O_9$	MS^2[633]:101.0233(100),113.0235(52),161.0450(17)	Eclalbasaponin A	Triterpenoids
144	18.78	805.40159	805.40350	2.366	$[M-H]^-$	$C_{42}H_{62}O_{15}$	MS^2[805]:113.0234(100),351.0575(35)	Licoricesaponin C2	Triterpenoids
145	18.93	367.11871	367.11938	1.821	$[M-H]^-$	$C_{21}H_{20}O_6$	MS^2[367]:309.0411(100),297.0410(33),203.0711(7),352.0953(2)	Glycycoumarin	Coumarins
146	18.98	807.41724	807.41919	2.41	$[M-H]^-$	$C_{42}H_{64}O_{15}$	MS^2[807]:113.0234(100),193.0351(50),351.0575(41),289.0574(6)	Licoricesaponin B2	Triterpenoids
147	19.04	305.17583	305.17645	2.203	$[M-H]^-$	$C_{18}H_{26}O_4$	MS^2[305]:135.0808(100),249.1500(52)	Octyl ferulate	Phenolic acids
148	19.09	311.12778	311.12677	-3.264	$[M+H]^+$	$C_{19}H_{18}O_4$	MS^2[311]:275.1057(100),293.1160(36), 265.1213(20),283.1318(14)	Tanshinone IIB	Quinones
149	19.47	315.08631	315.08505	-4.014	$[M+H]^+$	$C_{17}H_{14}O_6$	MS^2[315]:300.06177(100),315.08505(66)	Pectolinarigenin	Flavonoids
150	19.61	597.30453	597.30609	2.601	$[M-H]^-$	$C_{27}H_{51}O_{12}P$	MS^2[597]:281.2490(100),152.9951(60),241.0119(32)	1-(9Z-octadecenoyl)-sn-glycero-3-phospho-(1'-myo-inositol)	Other categories
151	19.64	355.11761	355.11633	-3.618	$[M-H]^-$	$C_{20}H_{18}O_6$	MS^2[355]:299.0539(100),355.1157(22),287.0542(6)	Licoflavonol	Flavonols
152	19.64	351.12379	351.12454	2.116	$[M-H]^-$	$C_{21}H_{20}O_5$	MS^2[351]:351.1243(100),336.1	Gancaonin M	Isoflavonoids

							010(87)		
153	19.68	297.11213	297.11118	-3.216	$[M+H]^+$	$C_{18}H_{16}O_4$	MS^2[297]:251.1058(100),223.1109(40),279.1005(31)	Danshenxinkun A	Triterpenoids
154	19.88	339.15908	339.15784	-3.673	$[M+H]^+$	$C_{21}H_{22}O_4$	MS^2[339]:121.0282(100),297.1474(9)245.1165(6),271.0955(5),339.1579(5),107.0491(4)	Licochalcone A	Isoflavonoids
155	20.11	309.11213	309.11102	-3.609	$[M+H]^+$	$C_{19}H_{16}O_4$	MS^2[309]:281.1162(100),263.1055(12),235.1106(5)	Salshenaldehyde	Phenolic acids
156	20.26	335.09249	335.09338	2.336	$[M-H]^-$	$C_{20}H_{16}O_5$	MS^2[335]:291.1032(17),320.0696(3),307.0989(2)	Glabrone	Isoflavonoids
157	20.46	269.04554	269.04610	2.205	$[M-H]^-$	$C_{15}H_{10}O_5$	MS^2[269]:269.0460(100),225.0558(4),241.0507(2)	Baicalein	Flavonoids
158	20.79	279.10157	279.10056	-3.622	$[M+H]^+$	$C_{18}H_{14}O_3$	MS^2[279]:261.0901(100),233.0953(80),279.1006(48),209.0954(10),169.0643(8),251.1059(5)	Dihydrotanshinone I	Quinones
159	21.01	339.12270	339.12152	-3.48	$[M+H]^+$	$C_{20}H_{18}O_5$	MS^2[339]:261.0900(100),233.0953(20),205.1004(2)	Methyltanshinonate	Quinones
160	21.11	393.20603	393.20447	-3.982	$[M+H]^+$	$C_{25}H_{28}O_4$	MS^2[393]:167.0333(100),149.0228(73),137.0590(3),177.0541(7),203.0696(17),337.1421(9)	Kanzonol C	Flavonols
161	21.76	302.30535	302.30405	-4.320	$[M+H]^+$	$C_{18}H_{39}NO_2$	MS^2[302]:302.3041(100),284.2936(12),88.0759(13),106.0862(11)	2,2'-(Tetradecylimino)diethanol	Other categories
162	21.83	468.30846	468.30676	-3.642	$[M+H]^+$	$C_{22}H_{46}NO_7P$	MS^2[468]:184.0727(100),104.1070(76),86.0966(22)	1-Myristoyl-sn-glycero-3-phosphocholine	Other categories

163	21.89	297.14852	297.14725	-4.277	$[M+H]^+$	$C_{19}H_{20}O_3$	MS^2[297]:297.1472(100),251.1419(27),279.1368(26),237.0904(4)	Cryptotanshinone	Quinones
164	22.18	277.08592	277.08493	-3.576	$[M+H]^+$	$C_{18}H_{12}O_3$	MS^2[277]:249.0900(100),277.0849(66),231.0797(10),221.0953(8)	Tanshinone I	Quinones
165	22.27	452.27826	452.27936	2.427	$[M-H]^-$	$C_{21}H_{44}NO_7P$	MS^2[452]:255.2333(100),196.0377(10),140.0109(3)	1-Palmitoyl-2-hydrOxy-sn-glycero-3-phosphoethanolamine	Other categories
166	22.56	293.11722	293.11618	-3.551	$[M+H]^+$	$C_{19}H_{16}O_3$	MS^2[293]:247.11092(100),275.10559(83),265.12143(18),251.06931(17)	Dehydrotanshinone II A	Quinones
167	22.76	281.15360	281.15265	-3.402	$[M+H]^+$	$C_{19}H_{20}O_2$	MS^2[281]:253.15781(100),221.09535(97),266.12918(64),238.13437(44)	Dehydromiltirone	Quinones
168	23.13	295.13280	295.13156	-1.31	$[M+H]^+$	$C_{19}H_{18}O_3$	MS^2[295]:277.1213(100),249.1263(56),235.0744(16),266.0927(13),262.0979(12),280.1087(11)	Tanshinone IIA	Quinones
169	23.34	283.16925	283.16824	-3.59	$[M+H]^+$	$C_{19}H_{22}O_2$	MS^2[283]:223.11093(100),265.1576(59),240.1133(35),254.1294(11)	Miltrione	Quinones
170	24.42	455.35306	455.35406	2.177	$[M-H]^-$	$C_{21}H_{20}O_6$	MS^2[455]:455.3541(100),456.3573(6)	Oleanolic acid	Triterpenoids

* Compared with standard compounds.

Table S2. Detailed information of the 22 reference standards.

Compound Name	Formula	Lot Number	Supplier
Isorhamnetin	$C_{16}H_{12}O_7$	Y-039-181103	Chengdu Ruifensi Biotechnology Co.,Ltd., Chengdu, China
Emodin	$C_{15}H_{10}O_5$	A10057	Shanghai Yuanye Biotechnology Co., Ltd., Shanghai, China
Citric acid	$C_6H_8O_7$	C108869	Tianjin Zhiyuan Chemical Reagent Co.,Ltd., Tianjin, China
Quercetin	$C_{15}H_{10}O_7$	C28J11Y116820	Shanghai Yuanye Biotechnology Co.,Ltd., Shanghai, China
Physcion	$C_{16}H_{12}O_5$	B20242	Shanghai Yuanye Biotechnology Co., Ltd., Shanghai, China
Liquiritigenin	$C_{15}H_{12}O_4$	AB0563-0020	Chengdu Efa Biotechnology Co., Ltd., Chengdu, China
3,5-Dicaffeoylquinic acid	$C_{25}H_{24}O_{12}$	Y-068-170903	Chengdu Ruifensi Biotechnology Co., Ltd., Chengdu, China
Procyanidin B1	$C_{30}H_{26}O_{12}$	wkq19062802	Sichuan Weikeqi Biotechnology Co.,Ltd., Sichuan, China
Rutin	$C_{27}H_{30}O_{16}$	AF8032520	Chengdu Efa Biotechnology Co., Ltd., Chengdu, China
Cryptochlorogenic acid	$C_{16}H_{18}O_9$	MUST-15011413	Chengdu Manste Biotechnology Co.,Ltd., Chengdu, China
Caffeic acid	$C_9H_8O_4$	C108306	Aladdin, Shanghai, China
Luteolin	$C_{15}H_{10}O_6$	M-007-190422	Chengdu Ruifensi Biotechnology

			Co.,Ltd., Chengdu, China
Malic acid	$C_4H_6O_5$	M105695	Spring Autumn, Nanjing, China
Sucrose	$C_{12}H_{22}O_{11}$	V59943	Shanghai Yuanye Biotechnology Co., Ltd., Shanghai, China
Rosmarinic acid	$C_{18}H_{16}O_8$	20060201	Chengdu Pufeide Biotechnology Co.,Ltd., Chengdu, China
4-hydroxybenzoic acid	$C_7H_6O_3$	DH1925000	Shandong Xiya Chemical Co.,Ltd., Shandong, China
D-Galactose	$C_6H_{12}O_6$	B21893	Shanghai Yuanye Biotechnology Co., Ltd., Shanghai, China
D-ribose	$C_5H_{10}O_5$	B21897	Shanghai Yuanye Biotechnology Co., Ltd., Shanghai, China
Puerarin	$C_{21}H_{20}O_9$	20111105	Chengdu Pufeide Biotechnology Co.,Ltd., Chengdu, China
Acetylshikonin	$C_{18}H_{18}O_6$	18060506	Chengdu Pufeide Biotechnology Co.,Ltd., Chengdu, China
Glycyrrhizic acid	$C_{42}H_{62}O_{16}$	100486660	Sigma-Aldrich, Shanghai, China
Guanine	$C_5H_5N_5O$	B20906	Shanghai Yuanye Biotechnology Co., Ltd., Shanghai, China

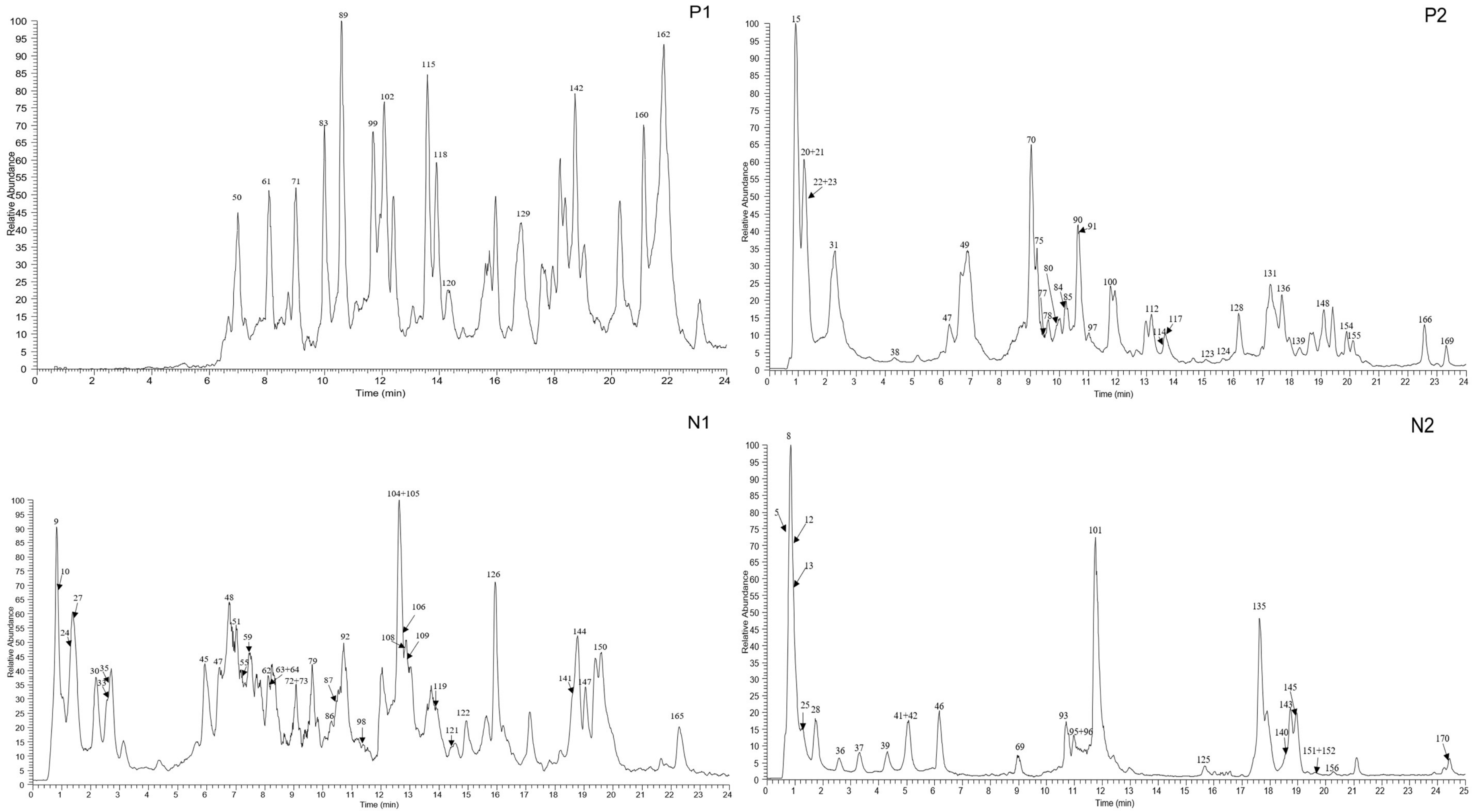

Figure S1. High-resolution extraction ion chromatograms (TIC) of XBKF capsules in positive-ion (P1,P2) and negative-ion (N1,N2) modes. P1 m/z152.05668, 193.04953, 209.08083, 220.11794, 271.06009, 277.08592, 281.15360, 285.07575, 295.09648, 295.13280, 297.11213, 297.14852, 301.07066,

302.30535, 315.04992, 319.04484, 339.10744, 393.20603, 417.11800, 417.33632, 419.13365, 433.11292, 468.30846, 551.10314, 551.17591, 565.15518; P2 m/z124.03930, 130.08589, 138.05495, 139.03897, 152.05668, 166.08625, 181.04953, 182.08116, 193.04953, 209.08083, 220.11794, 257.08083, 266.12342, 269.08083, 271.09648, 278.12342, 279.10157, 283.16824, 290.08813,293.11618, 297.11213, 309.11213, 311.12778, 315.08631, 317.06557, 319.04484, 328.13907, 339.10744, 339.12270, 339.15784, 339.15908, 341.06557, 344.13399, 355.10086, 431.13365, 431.31558, 433.11292, 433.33123, 441.20201, 449.10783, 465.10275, 469.33123, 471.34688, 492.31670, 517.13405, 539.11707, 539.11840, 556.21772, 611.16066, 654.36953, 1079.52687; N1 m/z147.02989, 149.04554, 151.04006, 161.02441, 163.04006, 167.03498, 173.08193, 177.01933, 177.04046, 193.05060, 193.05063, 201.11323, 209.03029, 218.10339, 253.05063, 255.06628, 259.02244, 271.06119, 283.06119, 285.04046, 285.07684, 289.07176, 292.14017, 299.05611, 301.03537, 303.05102, 305.17583, 307.07243, 313.07176, 315.05102, 329.08780, 329.10306, 337.09289, 353.13944, 373.09289, 417.08271, 433.11402, 445.11402, 447.09328, 449.10893, 452.27826, 461.10893, 479.11840, 521.13006, 577.13514, 593.15119, 597.30453, 609.14610, 625.14102, 805.40159; N2 m/z117.01933, 133.01424, 137.02441, 153.01933, 161.04554, 165.05571, 169.01424, 173.00916, 175.06119, 179.05611, 187.13396, 191.01972, 191.05611, 195.05102, 197.04554, 201.11323, 265.14790, 285.04046, 290.08813, 299.05611, 313.07176, 335.09249, 341.10893, 351.12379, 353.08780, 355.11761, 359.07724, 367.11871, 387.11441, 449.10893, 455.35306, 493.11402, 521.13006, 537.10384, 539.11949, 549.16136, 551.11949, 563.14062, 577.13514, 633.40080, 665.21458, 717.14610, 795.45362, 807.41724, 821.39650, 837.39142, 875.41044.
